# Targeting OGG1 arrests cancer cell proliferation by inducing replication stress

**DOI:** 10.1093/nar/gkaa1048

**Published:** 2020-11-19

**Authors:** Torkild Visnes, Carlos Benítez-Buelga, Armando Cázares-Körner, Kumar Sanjiv, Bishoy M F Hanna, Oliver Mortusewicz, Varshni Rajagopal, Julian J Albers, Daniel W Hagey, Tove Bekkhus, Saeed Eshtad, Juan Miguel Baquero, Geoffrey Masuyer, Olov Wallner, Sarah Müller, Therese Pham, Camilla Göktürk, Azita Rasti, Sharda Suman, Raúl Torres-Ruiz, Antonio Sarno, Elisée Wiita, Evert J Homan, Stella Karsten, Karthick Marimuthu, Maurice Michel, Tobias Koolmeister, Martin Scobie, Olga Loseva, Ingrid Almlöf, Judith Edda Unterlass, Aleksandra Pettke, Johan Boström, Monica Pandey, Helge Gad, Patrick Herr, Ann-Sofie Jemth, Samir El Andaloussi, Christina Kalderén, Sandra Rodriguez-Perales, Javier Benítez, Hans E Krokan, Mikael Altun, Pål Stenmark, Ulrika Warpman Berglund, Thomas Helleday

**Affiliations:** Science for Life Laboratory, Department of Oncology and Pathology, Karolinska Institutet, S-171 76 Stockholm, Sweden; Department of Biotechnology and Nanomedicine, SINTEF Industry, N-7465 Trondheim,Norway; Science for Life Laboratory, Department of Oncology and Pathology, Karolinska Institutet, S-171 76 Stockholm, Sweden; Science for Life Laboratory, Department of Oncology and Pathology, Karolinska Institutet, S-171 76 Stockholm, Sweden; Science for Life Laboratory, Department of Oncology and Pathology, Karolinska Institutet, S-171 76 Stockholm, Sweden; Science for Life Laboratory, Department of Oncology and Pathology, Karolinska Institutet, S-171 76 Stockholm, Sweden; Science for Life Laboratory, Department of Oncology and Pathology, Karolinska Institutet, S-171 76 Stockholm, Sweden; Science for Life Laboratory, Department of Oncology and Pathology, Karolinska Institutet, S-171 76 Stockholm, Sweden; Science for Life Laboratory, Department of Oncology and Pathology, Karolinska Institutet, S-171 76 Stockholm, Sweden; Department of Laboratory Medicine, Karolinska Institutet, Stockholm, Sweden; Science for Life Laboratory, Department of Oncology and Pathology, Karolinska Institutet, S-171 76 Stockholm, Sweden; Science for Life Laboratory, Department of Oncology and Pathology, Karolinska Institutet, S-171 76 Stockholm, Sweden; Human Genetics Group, Spanish National Cancer Research Centre (CNIO), Madrid, Spain; Department of Biochemistry and Biophysics, Stockholm University, SE-106 91 Stockholm, Sweden; Department of Pharmacy and Pharmacology, Centre for Therapeutic Innovation. University of Bath, Bath BA2 7AY, UK; Science for Life Laboratory, Department of Oncology and Pathology, Karolinska Institutet, S-171 76 Stockholm, Sweden; Science for Life Laboratory, Department of Oncology and Pathology, Karolinska Institutet, S-171 76 Stockholm, Sweden; Science for Life Laboratory, Department of Oncology and Pathology, Karolinska Institutet, S-171 76 Stockholm, Sweden; Science for Life Laboratory, Department of Oncology and Pathology, Karolinska Institutet, S-171 76 Stockholm, Sweden; Science for Life Laboratory, Department of Oncology and Pathology, Karolinska Institutet, S-171 76 Stockholm, Sweden; Science for Life Laboratory, Department of Oncology and Pathology, Karolinska Institutet, S-171 76 Stockholm, Sweden; Molecular Cytogenetics Group, Human Cancer Genetics Program, Spanish National Cancer Research Centre (CNIO), Madrid, 28029, Spain; Josep Carreras Leukemia Research Institute and Department of Biomedicine, School of Medicine, University of Barcelona, Barcelona 08036, Spain; Department of Clinical and Molecular Medicine, Norwegian University of Science and Technology, Trondheim, Norway; The Liaison Committee for Education, Research and Innovation in Central Norway, Trondheim, Norway; Department of Environment and New Resources, SINTEF Ocean, N-7010 Trondheim, Norway; Science for Life Laboratory, Department of Oncology and Pathology, Karolinska Institutet, S-171 76 Stockholm, Sweden; Science for Life Laboratory, Department of Oncology and Pathology, Karolinska Institutet, S-171 76 Stockholm, Sweden; Science for Life Laboratory, Department of Oncology and Pathology, Karolinska Institutet, S-171 76 Stockholm, Sweden; Science for Life Laboratory, Department of Oncology and Pathology, Karolinska Institutet, S-171 76 Stockholm, Sweden; Science for Life Laboratory, Department of Oncology and Pathology, Karolinska Institutet, S-171 76 Stockholm, Sweden; Science for Life Laboratory, Department of Oncology and Pathology, Karolinska Institutet, S-171 76 Stockholm, Sweden; Science for Life Laboratory, Department of Oncology and Pathology, Karolinska Institutet, S-171 76 Stockholm, Sweden; Science for Life Laboratory, Department of Oncology and Pathology, Karolinska Institutet, S-171 76 Stockholm, Sweden; Science for Life Laboratory, Department of Oncology and Pathology, Karolinska Institutet, S-171 76 Stockholm, Sweden; Science for Life Laboratory, Department of Oncology and Pathology, Karolinska Institutet, S-171 76 Stockholm, Sweden; Science for Life Laboratory, Department of Oncology and Pathology, Karolinska Institutet, S-171 76 Stockholm, Sweden; Science for Life Laboratory, Department of Oncology and Pathology, Karolinska Institutet, S-171 76 Stockholm, Sweden; Science for Life Laboratory, Division of Clinical Physiology, Department of Laboratory Medicine, Karolinska Institutet, Stockholm, Sweden; Weston Park Cancer Centre, Department of Oncology and Metabolism, University of Sheffield, Sheffield S10 2RX, UK; Weston Park Cancer Centre, Department of Oncology and Metabolism, University of Sheffield, Sheffield S10 2RX, UK; Weston Park Cancer Centre, Department of Oncology and Metabolism, University of Sheffield, Sheffield S10 2RX, UK; Science for Life Laboratory, Department of Oncology and Pathology, Karolinska Institutet, S-171 76 Stockholm, Sweden; Department of Laboratory Medicine, Karolinska Institutet, Stockholm, Sweden; Science for Life Laboratory, Department of Oncology and Pathology, Karolinska Institutet, S-171 76 Stockholm, Sweden; Molecular Cytogenetics Group, Human Cancer Genetics Program, Spanish National Cancer Research Centre (CNIO), Madrid, 28029, Spain; Human Genetics Group, Spanish National Cancer Research Centre (CNIO), Madrid, Spain; Spanish Network on Rare Diseases (CIBERER), Madrid, Spain; Department of Clinical and Molecular Medicine, Norwegian University of Science and Technology, Trondheim, Norway; The Liaison Committee for Education, Research and Innovation in Central Norway, Trondheim, Norway; Science for Life Laboratory, Department of Oncology and Pathology, Karolinska Institutet, S-171 76 Stockholm, Sweden; Science for Life Laboratory, Division of Clinical Physiology, Department of Laboratory Medicine, Karolinska Institutet, Stockholm, Sweden; Department of Biochemistry and Biophysics, Stockholm University, SE-106 91 Stockholm, Sweden; Department of Experimental Medical Science, Lund University, SE-221 00 Lund, Sweden; Science for Life Laboratory, Department of Oncology and Pathology, Karolinska Institutet, S-171 76 Stockholm, Sweden; Science for Life Laboratory, Department of Oncology and Pathology, Karolinska Institutet, S-171 76 Stockholm, Sweden; Weston Park Cancer Centre, Department of Oncology and Metabolism, University of Sheffield, Sheffield S10 2RX, UK

## Abstract

Altered oncogene expression in cancer cells causes loss of redox homeostasis resulting in oxidative DNA damage, e.g. 8-oxoguanine (8-oxoG), repaired by base excision repair (BER). PARP1 coordinates BER and relies on the upstream 8-oxoguanine-DNA glycosylase (OGG1) to recognise and excise 8-oxoG. Here we hypothesize that OGG1 may represent an attractive target to exploit reactive oxygen species (ROS) elevation in cancer. Although OGG1 depletion is well tolerated in non-transformed cells, we report here that OGG1 depletion obstructs A3 T-cell lymphoblastic acute leukemia growth *in vitro* and *in vivo*, validating OGG1 as a potential anti-cancer target. In line with this hypothesis, we show that OGG1 inhibitors (OGG1i) target a wide range of cancer cells, with a favourable therapeutic index compared to non-transformed cells. Mechanistically, OGG1i and shRNA depletion cause S-phase DNA damage, replication stress and proliferation arrest or cell death, representing a novel mechanistic approach to target cancer. This study adds OGG1 to the list of BER factors, e.g. PARP1, as potential targets for cancer treatment.

## INTRODUCTION

Transformed cancer cells are characterized by increased levels of DNA damage (1,2) as a result of lost redox homeostasis, oncogene-induced replication stress and impaired DNA repair pathways (3,4). As a consequence, cancers may become addicted to efficient repair and hence, targeting the DNA damage response and repair (DDR) pathways is an established anti-cancer strategy (5). The base excision repair (BER) pathway, involving PARP1 and glycosylases such as 8-oxoguanine-DNA glycosylase 1 (OGG1), is repairing the vast majority of DNA lesions. We and others previously demonstrated that PARP inhibitors are effective in treatment of homologous recombination defective (HRD) cancers (6,7) and currently four different PARP inhibitors are approved for treating several HRD cancers. By trapping PARP on DNA (8), clinically used PARP inhibitors interfere with replication forks (9,10), alternative end-joining (11–13) and other pathways (14,15). Whereas PARP1-inhibitors also blocks BER (16), targeting the BER pathway as such has not been validated as an anti-cancer target. Most of the focus in the DDR inhibitor (DDRi) field has been targeting proteins involved in signalling DNA double-strand breaks (DSBs) or replication stress (RS), such as ATR, ATM, CHK1 and WEE1 kinases currently evaluated in clinical trials (17).

It is well established that expression of oncogenes such as *MYC* and *RAS* lead to the generation of reactive oxygen species (ROS) and oxidative DNA damage (18–21). Thus, high levels of oxidized bases have been found in the genome of cancer cells (22,23), which excrete oxidized bases and nucleotides into serum and urine serving as robust biomarkers for cancer (22,23), also reviewed in (24,25). The most common result of ROS DNA damage is the oxidation of guanine to 8-dihydro-7,8-oxoguanosine (8-oxodG) in DNA, repaired by OGG1. Whereas the presence of 8-oxodG in DNA is miscoding, the signature C→A transversion mutation is surprisingly rare in human malignancies (26). This indicates that high-ROS cancers may rely on efficient pathways to repair ROS-induced DNA damage.

Surprisingly, *Ogg1^−^^/^^−^* mice are alive and grow old, albeit having increased incidence of lung cancer at the age of 18 months (27). Interestingly, OGG1 overexpression protects cells against Ras-induced senescence (28) and high OGG1 expression is correlated with lower genomic instability in a panel of adenocarcinoma cell lines (29). Moreover, the transcriptional activity of genes (*PCNA*, *KRAS*, *MYC*, VEGF) and transcription factors (NF-κB) involved in cell proliferation, and initiation or progression of cancer can be modulated upon introduction and processing of 8-oxodG within promoters and 5′ untranslated regions via BER (30–34), altogether suggesting a role for OGG1 in cancer development.

While targeting the BER pathway, with for instance PARP inhibitors, is a validated strategy to treat cancer, other BER targeting strategies have received surprisingly little attention (35,36). Here, we validate OGG1 as an anti-cancer target, which confirms targeting oxidative DNA repair as a concept for treatment of cancer. Furthermore, this validates that BER inhibitors, other than PARP inhibitors, are effective as anti-cancer treatments.

## MATERIALS AND METHODS

### Cell culture

Adherent and suspension cell lines were cultured in RPMI (61870-010 Thermo Fisher Scientific), McCoy's (36600-021 Thermo Fisher Scientific) or DMEM (10566-016 Thermo Fisher Scientific) media depending on the cell line. The media was supplemented with 10% fetal bovine serum (10500064, Thermo Fisher Scientific) and 100 U/ml Penicillin Streptomycin (15140122, Thermo Fisher Scientific) and the cells were cultured at 37°C and 5% carbon dioxide. The BJ-Tert and BJ-Ras cell lines were provided by W. Hahn (Dana-Farber Cancer Institute), MEF *Ogg1*^−/-^ cells from M. Bignami (Istituto Superiore di Sanità, Rome, Italy), HCT116 and HCT116+Chr3 human colon carcinoma cells were obtained from Dr. Bert Vogelstein (2001, Johns Hopkins, Baltimore, MD, USA), Hec59 and Hec59+Chr2 from (37), LCL#1 and LCL#2 from J. Benitez (Spanish National Cancer Research Centre, Madrid, Spain), and the rest of the cell lines were sourced from commercial suppliers American Type Culture Collection (ATCC) or the German Collection of Microorganisms and Cell Cultures GmbH (DMSZ). All cultures were passaged a maximum of 25 times after thawing from stock vials and checked for mycoplasma contamination using MycoAlert™ Mycoplasma Detection Kit (Lonza) every other month.

### RNA interference

200 000 immortalized BJ-Tert and 100 000 transformed BJ-Ras-SV40 cells were seeded in six-well plates, incubated overnight and transfected with siRNA duplexes using Interferin (# 409–50, Polyplus Transfection) according to instructions. After 48 h, 3000 BJ-Tert cells and 1000 BJ-Ras cells per well were seeded in 96-well plates and reverse transfected with the same siRNA sequence according to instructions. The final concentrations of siRNA duplexes were 10 and 2.7 nM for the forward and reverse transfections, respectively, and the siRNA sequences were siOGG1#1: 5′-GGAUCAAGTAUGGACACUGAC-3′, siOGG1#2: 5′-GGACAAUCUUUCCGGUGGA-3′. AllStars negative control siRNA (SI03650318, Qiagen) was used as transfection control.

Stable transfections with doxycycline-inducible small hairpin RNA was performed with the plasmid pRSITEP-U6Tet-(sh)-EF1-TetRep-P2A-Puro-P2A-RFP670 (38) and transduced into A3 and H460 cell lines as described (38). The non-targeting shRNA sequence was identical to the one described in (38), and the hairpins recognizing OGG1 hybridized to the following sequences in the OGG1 open reading frame shOGG1#1: 5′-GGAGTGGTGTACTAGCGGATC-3′, shOGG1#2: 5′-GTGTGCGACTGCTGCGACAAG-3′ and shOGG1#3: 5′-TGTGCCCGTGGATGTCCATAT-3′.

### Viability

Cells were seeded in 96- or 384-well plates and incubated for 3 days for combination experiments or 5 days for single-drug exposure experiments. Resazurin (R7017, SigmaAldrich) was added to a final concentration of 0.01 mg/ml resazurin and fluorescence was measured at ex530/em590 after incubation for 2, 4 or 6 h. Curves were fitted using XLfit software (IDBS) or Prism 8.0 (Graphpad Software), and EC50 values were determined.

### Colony formation assays

BJ-Tert and BJ-Ras cell lines were transfected with siRNA for 48 h as described above, trypsinized, counted and seeded in 6-well plates at 300 cells per well and incubated until colony size surpassed 50 cells, followed by medium removal and addition of 4% (w/v) methylene blue in methanol. Following extensive washes in tap water and air drying, colonies with >50 cells were counted.

H460 cells harboring knockdown constructs were treated with 500 ng/ml doxycycline for 48 h, trypsinized, counted and seeded in 6-cm diameter petri dishes for 9 days in the presence of an equivalent concentration doxycycline. Colonies exceeding 50 cells were fixed, stained and enumerated as above.

The indicated concentrations TH5487 were spotted in 6-well plates using a Tecan D300e digital dispenser. 200–500 cells/well H460, ACHN, *Ogg1*^−/−^ mouse embryonic fibroblasts and MRC5 cells were then seeded at 200–500 cells/well and incubated for 7–11 days. Colonies exceeding 50 cells were fixed, stained and enumerated as above.

250 HEK293T or HEK293T (KO) cells were counted and seeded in 6-well plates for 8 days in the presence or absence of TH5487 at indicated concentrations. Colonies were directly scanned after removing media and drying colonies. Area of HEK293T or HEK293T(KO) colonies was digitally colour contrasted and then analysed using ImageJ.

### PBMC isolation

Peripheral blood mononuclear Cells (PBMC) were isolated from peripheral whole blood from controls using Histopaque^®^-1077 (Sigma-Aldrich, San Luis, MO, USA) following the manufacturer's instructions. Briefly, peripheral blood was collected in heparin and diluted with equal amount of PBS. Next, blood was centrifuged with Histopaque^®^-1077 at 400 × *g* for 30 min at room temperature and the PBMC layer was recovered. All steps were processed within 4 h after blood extraction.

The samples were obtained from healthy donors who signed an appropriate informed consent and the proposal was approved by the ethics committee at the Fuenlabrada University Hospital, Madrid, Spain. The study was performed in accordance with the principles of the Declaration of Helsinki.

### CD34+ isolation and culture

Isolation of total CD34+ cells was performed from umbilical cord blood samples (CB) from healthy donors distributed from Centro de Transfusión de la Comunidad de Madrid. All samples were collected under written consent and institutional review board agreement. CD34+ cells was obtained from mononuclear cells were separated by fractionation in Ficoll-hypaque according to manufacturer's recommendations (GE Healthcare). Purified CD34+ cells were obtained using a MACS CD34 Micro-Bead kit (Miltenyi Biotec) and were cultured in StemSpan SFM II (StemCell Technologies) containing 100 U/ml penicillin/streptomycin (Gibco) and a cytokine cocktail of SCF (100 ng/ml), TPO (100 ng/ml), Flt3 ligand (100 ng/ml, Peprotech. Cells were cultured at 37°C, 5% CO_2_ and 5% O_2_.

### Activation of T-cells using Phytohemagglutinin-L (PHA-L) or dynabeads

PMBCs and CD34- fraction (CB) were cultured in the presence of PHA-L (Sigma-Aldrich, ref: 11249738001) or Dynabeads^®^ Human T-Activator CD3/CD28 (Thermofisher scientific, ref: 11131D) for the activation and expansion of human T cells according to the manufacturer's instructions.

### CD3 flow cytometry assay

The experiment was performed on blood cells from 4 different healthy individuals, with three replicates each. Human peripheral blood mononuclear cells were isolated from fresh buffy coats obtained from healthy donors via the Karolinska Hospital, Stockholm, Sweden. For separation, Ficoll-Paque PLUS density medium (17144003, GE Healthcare) and SepMate separation tubes (85450, StemCell) were used, according to manufacturer's instructions. Briefly, buffy coat diluted 1:1 with PBS and layered on 15 ml of Ficoll-Paque PLUS in the SepMate tubes was spun down for 10 min at 12 000 × g. The upper layer of the tube content was then poured into new falcon tubes and washed twice with PBS.

Cells were seeded out in round bottom 96-well plates (83.3925.500, Sarstedt) in RPMI Medium 1640 containing GlutaMAX™ (61870-010, ThermoFisher) supplemented with 10% human AB+ male heat inactivated clotted whole blood serum (H5667, Sigma-Aldrich) and 100 U/ml penicillin/streptomycin (15140122, Gibco). Non-activated cells were seeded at a concentration of 1 × 10^6^ cells/ml. For CD3/CD28 activation, Dynabeads™ Human T-Activator CD3/CD28 (11131D, ThermoFisher) were mixed with 0.8 1 × 10^6^ cells/ml at a concentration of 0.75 beads per cell. Only viable cells were counted, using a TC20™ automated cell counter (Bio-Rad) and Trypan blue (1450021, Bio-Rad). The total volume of medium per well was 200 μl. DMSO and TH5487 were added directly into the wells, at a concentration of 0.25% and 25 μM, respectively. After 3 days of activation and treatment, 50 μl fresh complete medium containing DMSO or TH5487 was added to the wells for an additional 3 days.

After 6 days of culture, 90 μl cells were moved to a Nunc™ 96-Well Polystyrene Conical Bottom MicroWell™ Plate (249935, ThermoFisher), containing 10 μl of cold CellWASH (349524, BD) per well, supplemented with 5 μl Precision Count Beads™ (424902, BioLegend) and 0.5 μl of each of the following antibodies: PE-Cy™7 Mouse Anti-Human CD3 (563423, BD), PE Mouse Anti-Human CD8 (561950, BD), APC Mouse Anti-Human CD71 (334108, Nordic Biosite) as an additional activation marker and APC-H7 Mouse Anti-Human CD4 (560158, BD). The plate was incubated at 4°C for 30 min and then washed with cold CellWASH. The samples were then resuspended in 100 μl of Annexin V Binding buffer (422201, Nordic Biosite), containing 0.5 μl FITC-conjugated Annexin V (556420, BD) and 1:500 SYTOX™ Blue Dead Cell Stain (S34857, ThermoFisher) and incubated at RT for 15 min, protected from light. The cells were then moved to flow cytometry tubes containing 100 μl additional CellWASH, incubated on ice for at least 10 min and then run with a 3-laser Navios flow cytometer from Beckman Coulter.

10 000 events from an FSC-SSC gate set on the typical living lymphocyte population were collected and the data was compensated and analyzed using FlowJo 10.5.3. The CD3 positive cells were gated for and plotted in a Sytox Blue-Annexin V graph, where the double-negative cells were considered viable. The number of CD3 positive, viable events was quantified and then divided by the number of Precision Count Beads™ collected per tube. This cell-per-bead number was further divided by the mean of the DMSO viable CD3+ cell-per-bead population, in order to generate a relative concentration of living CD3 positive cells in relation to the DMSO samples. For gating, Fluorescence-Minus-One (FMO) samples were used as technical controls. For compensation, single-stains and unstained samples were run fresh for every new experiment. Data was further analyzed and plotted using Microsoft Excel and GraphPad Prism 8.

### Lentiviral generation of cell lines

The *Photinus pyralis* luciferase gene from the vector pGL4.32 (Promega) was cloned into pENTR1A no CCDB plasmid (w48-1, AddGene 17398), and shuttled into the pLenti-PGK-hygro DEST (w530-1, AddGene 19066) plasmid, packaged into lentiviruses as described (38) and transduced into A3 cells containing shRNA constructs as described above and selected with 300 μg/ml hygromycin or 20 μg/ml blastidicin for 10 days.

### Immunoblotting

Cells were washed in cold PBS and lysed in RIPA buffer (50 mM Tris–HCl pH 7.5, 150 mM NaCl, 1 mM EDTA, 1% NP-40, 0.5% sodium deoxycholate and 0.1% SDS supplemented with complete protease inhibitor cocktail (04693116001, Roche), freeze-thawed once, incubated 20 min on ice followed by sonication at 80% amplitude, 0.7 cycle and 10 cycles in a UP100H (Hielscher Ultrasonics) and clarification by centrifugation. Proteins were separated and blotted with 4–12% polyacrylamide gels and the Trans-Blot Turbo transfer system, respectively (BioRad). The following primary antibodies were used: rabbit anti-OGG1 (ab124741, Abcam) 1:2500, mouse anti-Actin (ab6276, Abcam) 1:10 000, goat anti-vinculin 1:1000, rabbit anti-histone 3 (ab1791, Abcam) 1:5000, mouse anti-γH2AX(p-Ser139) (05-636, Millipore) 1:2000).

### 8-oxodG detection in genomic DNA

8-oxodG detection in genomic DNA through LC–MS/MS (39) and single-cell electrophoresis (40) were performed as described.

### Immunofluorescence

A3 cells were seeded in a 96-well plate 24 h prior to start of the experiment. Cells were then treated with 10 μM TH5487, for 24, 48 and 72 h, respectively or with 2 mM hydroxyurea for 1 h. Final DMSO concentration was 0.2%. After the treatment, cells were transferred to an imaging plate (black Falcon 96-well plate) pre-coated with 0.1% poly l-lysine (Sigma). Cells were allowed to attach for 15 minutes and were then fixed with 4% paraformaldehyde. The plate was washed with 0.05% Tween 20 in PBS, cells were permeabilized with 0.5% Triton-X100 in PBS and blocked with 2% bovine serum albumin in PBS. Incubation with primary antibody (mouse anti-γH2AX, Millipore, product no. 05-636, dilution 1:1000) in 2% BSA/PBS was performed at 4°C overnight. After washing with 0.05% Tween 20 in PBS, cells were incubated with secondary antibody (Alexa Fluor anti-mouse 647, Thermo Scientific, dilution 1:400) together with DAPI staining at room temperature for 1 h. Plate was washed thoroughly with 0.05% Tween 20 in PBS and imaged with ImageXpress (Molecular Devices, CA, USA). Image analysis was completed with Cell profiler software. Mean intensities of γH2AX signal in single nuclei were averaged and compared between the treatment and control. Statistical analysis was performed using GraphPad 7.0c software. Data from three independent experiments were pooled and analyzed for statistical significance by one-way analysis of variance.

### RNA-sequencing in A3 shNT cells

To extract bulk RNA from cells, the pellets were resuspended in 500 μl of TRI Reagent. After 5 min, 100 μl of chloroform was added and the tubes were shaken by hand for one minute. After 15 min incubation, the samples were centrifuged at 12 000 × *g* for 15 min at 4°C. 300 μl of the aqueous phase were then mixed thoroughly with 300 μl of Isopropanol, 30 μl of 3M Sodium Acetate and 1 μl of Pellet Paint (Merck 69049) and incubated over night at −20°C. The following day, the samples were centrifuged at 20 000 × *g* g for 30 min and the pellets washed twice with 600 μl of 70% ethanol. After drying, the pellets were resuspended in 15 μl of Elution Buffer and the concentration of RNA was measured using the Qubit RNA High Sensitivity Assay (Thermo Fischer Q32852) according to the manufacturer's instructions.

After diluting the samples, 2 ng of RNA were used as input for the Smart-seq2 RNA-sequencing protocol (41) and 50 bp single ends were sequenced on an Illumina HiSeq 3000 sequencer. Reads were mapped to the ENSEMBL human transcriptome GRCh37 using Tophat 2.1.1 to generate the read count matrix.

### Transcriptome data analysis in A3 cells

Differential expression analysis was performed with DESeq2 (v. 1.26.0) (42). DEGs were visualized using the EnhancedVolcano package (v.1.4.0). For functional analysis Gene Sets were retrieved with the msigdbr package (v7.1.1). For Gene Set Enrichment Analysis only genes with a mean expression >5 normalized counts as determined by DESeq2 were taken into account. GSEA was performed using the gsea package (v.1.12.0) that implements the algorithms described by Subramanian *et al.* (43). Heatmaps were generated using the ComplexHeatmap package (v.2.2.0) (44). Mapping of log_2_ fold normalized counts onto the DNA Replication KEGG (Kyoto Encyclopedia of Genes and Genomes) pathway was done using the pathview package (v.1.26.0) (45). All analyses were performed in R v.3.6.0.

### RNA extraction, quantification and real time PCR analysis

RNA was extracted from cultured cells using TRIzol^®^ Reagent (Thermo Fisher Scientific). RNA quantity and quality were assessed by NanoDrop® (ND-1000 V3.7.1; Thermo Fisher Scientific). The High-Capacity cDNA Reverse Transcription Kit (Applied Biosystems) was utilized for cDNA synthesis following the manufacturer's instructions. Two microliters of cDNA at a final concentration of 10 ng/μl were mixed with GoTaq^®^ qPCR MasterMix 1× (Promega) and 1 μM cDNA primers of each pair of primers (F/R) in a final volume reaction of 10 μl. Primers used are listed in Supplementary Table S2. The amplification conditions consisted of an initial step at 95°C for 10 min, followed by 40 cycles of 10 s at 95°C and 1 min at 60°C. Each qPCR was performed in duplicates or triplicates including no-template controls in an Abi QuantStudio 1 Flex Real-Time PCR System (Applied Biosystems). Relative transcript levels for each replication gene, was calculated using the 2ΔΔCt method for qPCR analysis after normalization with the housekeeping gene beta-actin (B-actin-F 5′-CCTGGCACCCAGCACAAT- 3′; B-actin-R 5′ - GGGCCGGACTCGTCATACT-3′).

### Oxidative DNA damage within MCM4 gene promoter

We adapted the procedure described by O’Callaghan *et al.* (46) to measure oxidative DNA damage at the *MCM4* gene promoter region. This is a qPCR method based on differences in PCR kinetics between DNA template digested by FPG and undigested DNA. We have modified the original protocol to incorporate another purified human OGG1 to quantify the accumulation of base lesions specific for OGG1, 8-oxoguanine. Conditions used for incubation were 200 ng of gDNA together with 2.4 μM of hOGG1 during 4 h. The quantitative real-time amplification of genomic DNA was performed as described by O’Callaghan *et al.* (46). Specific primers were used at a final concentration of 100 nM (*promoter*MCM4-F: 5′-GCTGTGATTGGTGAGGCCC -3′; *promoter*MCM4-R: 5′-CAAACCGCGAGACCCAGAG-3′) . Amplification cycling conditions were 10 min at 95°C, followed by 40 cycles of 95°C for 15 s and 60°C for 1 min. ΔCT method was run in an ABI quant studio 1 and all samples were loaded and analyzed in triplicate.

### EdU flow cytometry analysis

At the indicated time points, compound/dox treated cells were incubated in medium containing 10 μM 5-ethynyl-2′-deoxyuridine (EdU) (Thermo Fisher, A10044) for 20 min. Next, cells were fixed in cold 70% ethanol overnight. The azide alkyne Huisgen cycloaddition (‘Click reaction’) was carried out according to the Click-iT EdU Imaging Manual (Thermo Fisher) using various fluorescent dyes: Alexa Fluor® 488 azide (Thermo Fisher, A10266), Alexa Fluor^®^ 647 azide (Thermo Fisher, A10277), or TAMRA azide (Thermo Fisher, T10182), depending on assay conditions. Samples were incubated with Alexa Fluor^®^ 488 Mouse anti-H2AX (pS139) (BD Biosciences, 560445) overnight at 4°C and finally stained with DAPI solution [10 μg/ml DAPI (Sigma, D9542), 0.1 mg/ml RNase A (Thermo Fisher, EN0531), 1% BSA in PBS] for 20 min at room temperature. Measurements were done using a Navios Flow Cytometer (Beckman Coulter). Data was analyzed using Kaluza^®^ Flow Analysis Software (Beckman Coulter).

### Annexin V-staining

The annexin V staining experiment was performed using BD Pharmingen FITC Annexin V Apoptosis Detection Kit I (cat.no 556547) according to the protocol given in the kit. Cells were washed with ice-cold PBS and then washed with 1X Annexin buffer diluted in ice-cold PBS. To the washed pellet of cells, PI and FITC Annexin V dye was added and incubated at room temperature in dark for 15 mins. After the incubation, 400 µl of annexin buffer was added and run in Navios flow cytometer (Beckman Coulter) to detect the apoptosis with blue laser(488 nm, filter 525/40 nm) and dead cells with blue laser(488 nm, filter 620/30 nm). Kaluza Flow Analysis (Beckman Coulter) was used for data analysis.

### DNA fibre assay

A3 Cells were exposed to either 0.1% DMSO or 10 μM TH5487 for the indicated times, pulse-labeled with 25 μM 5-chloro-2′-deoxyuridine (CldU) for 30 min, washed with medium and pulse-labeled with 250 μM 5-iodo-2′-deoxyuridine (IdU) for 30 min. Alternatively, control or *OGG1* shRNA expression was induced with 1 μg/ml doxycyline for 48 h or 96 h before pulse labelling. CldU was detected by incubating acid-treated fiber spreads in Ibidi μ-Slide VI 0.4 (Ibidi, 80606) with rat anti-BrdU monoclonal antibody (AbD Serotec; cat# MCA2060), whereas IdU was detected using mouse anti-BrdU monoclonal antibody (BD Biosciences; cat# 347580) for 1 h at 37°C. Slides were fixed with 4% paraformaldehyde and incubated with goat anti-rat Alexa Fluor 555 or goat anti-mouse Alexa Fluor 488 for 1.5–2 hours. Fibers were examined using a Zeiss (Jena, Germany) LSM780 confocal laser scanning microscope with a 63× oil immersion objective. The lengths of red (AF 555) or green (AF 488) labeled patches were measured using the ImageJ software (National Institutes of Health; http://rsbweb.nih.gov/ij/) and arbitrary length values were converted into micrometers.

### Mouse xenograft experiment

Animal experiments were conducted as per the European directive, ethical guideline and regulations of the regional animal ethical committee Stockholm 2010/63 (N8914). Upon arrival to animal facility, animals were acclimatized in the animal house for a week with ad lib food and water, with a 12 h light cycle and the temperature and humidity set according to laboratory animal guidelines and regulations. For the xenograft experiment involving inducible shRNA, 1 × 10^7^ non-targeting luciferase transfected A3 cells in 50% matrigel in PBS were injected into flank region of BALB/c nude mice. Later, mice from non-targeting and shRNA groups were randomly divided into two groups containing eight mice per group. One group from both non-targeting and *OGG1*-targeting shRNA was given 1 mg/ml doxycycline in drinking water from day 15 till last survival of mice (when tumor size was about to reached 1000 mm^3^). For the experiment involving native A3 cells, 8 × 10^6^ luciferase-transfected cells in 50% matrigel were injected subcutaneously into NOD-SCID mice. Treatment was initiated 14 days after inoculation when tumour volume reached approximately 100 mm^3^. TH5487 was formulated in a vehicle solution of 5% DMSO, 10% tween-80, 10% Cremophor and 75% water, and the indicated doses were administered by oral gavage twice daily on weekdays for 6 weeks or until tumor size reached 1000 mm^3^. Tumor volume was measured using calipers (length × 0.52 × width × width) and bioluminescence images were taken using an IVIS spectrum *in vivo* imaging system (Perkin-Elmer) after intraperitoneal injection of D-luciferin (150 mg/kg in PBS). Following termination, tumors were ground in liquid nitrogen and resuspended in tris-buffered saline with complete protease inhibitors (Roche), homogenized and subjected to the indicated temperatures (47). Following lysis by three freeze-thaw cycles, samples were centrifuged at 20 000 × *g* for 30 min, and OGG1 in the supernatant was detected by immunoblotting. Survival plots were drawn using Graphpad Prism software.

### Biochemical assay

OGG1 activity was assessed *in vitro* as previously described (39).

### Synthetic chemistry experimental section

All reagents and solvents were purchased from Sigma-Aldrich, Combi-Blocks, Thermo Fischer Scientific, or VWR and were used without purification. The compound 7-bromo-3-(4-piperidyl)-1H-benzimidazol-2-one;2,2,2-trifluoroacetic acid was synthesized as described (39). Unless otherwise stated, reactions were performed without care to exclude air or moisture. Analytical thin-layer chromatography was performed on silica gel 60 F-254 plates (E. Merck) and visualized under a UV lamp. Flash column chromatography was performed in a Biotage^®^ SP4 MPLC system using Merck silica gel 60 Å (40–63 μm mesh). ^1^H and ^13^C NMR spectra were recorded on a Bruker DRX-400 MHz spectrometer. Chemical shifts are expressed in parts per million (ppm) and referenced to the residual solvent peak. Analytical LC–MS were performed on an Agilent MSD mass spectrometer connected to an Agilent 1100 system with: Method ST1090A3: Column ACE 3 C8 (50 × 3.0 mm); H_2_O (+ 0.1% TFA) and MeCN were used as mobile phases at a flow rate of 1 ml/min, with a gradient from 10–90% in 3 min; or Method B0597 × 3: Column Xterra MSC18 (50 × 3.0 mm); H_2_O (containing 10 mM NH_4_HCO_3_; pH 10) and MeCN were used as mobile phases at a flow rate of 1 ml/min, with a gradient of 5–97% in 3 min. For LC–MS, detection was made by UV (254 or 214 nm) and MS (ESI^+^). Preparative LC was performed on a Gilson system using Waters C18 OBD 5 μm column (30 × 75 mm) with water buffer (50 mM NH_4_HCO_3_ at pH 10) and acetonitrile as mobile phases using a flow rate of 45 ml/min. All final compounds were assessed to be >95% pure by LC–MS analysis.

TH5487 was synthesized as described (39).

*N*-(3,4-Dichlorophenyl)-4-[4-[6-(hydroxymethyl)-3- pyridyl]-2-oxo-3H-benzimidazol-1-yl]piperidine-1-carbox amide (TH5796).

Step 1: A mixture of 7-bromo-3-(4-piperidyl)-1H-benzimidazol-2-one;2,2,2-trifluoroacetic acid (1200 mg, 3.0 mmol), diisopropylethylamine (0.52 ml, 3.0 mmol), and 3,4-dichlorophenylisocyanate (560 mg, 3.0 mmol) in DCM (20 ml) was stirred at 20°C for 16 h. The product percipitated and was collected by filtration and washed with DCM, water, and then DCM again. The solid was dried at 40°C under vacuum. LCMS [M+H]^+^ 483.

Step 2: A mixture of 4-(4-bromo-2-oxo-3H-benzimidazol-1-yl)-*N*-(3,4-dichlorophenyl)piperidine-1-carboxamide (73 mg, 0.15 mmol), potassium carbonate (2M, 0.38 ml), [6-(hydroxymethyl)-3-pyridyl]boronic acid (69 mg, 0.45 mmol), and Pd(PPh_3_)_4_ (8.7 mg, 0.0075 mmol) was stirred under nitrogen atmosphere in dioxane at 95°C for 16 h. The mixture was thereafter concentrated and purified by preparative LC, the product was isolated as the TFA salt.

LCMS [M+H]^+^ 512. ^1^H NMR (400 MHz, DMSO-*d*_6_) δ ppm 11.22 (s, 1 H), 8.90 (s, 1 H), 8.79 (d, *J* = 1.6 Hz, 1 H), 8.30 (dd, *J* = 8.2, 1.9 Hz, 1 H), 7.89 (t, *J* = 1.3 Hz, 1 H), 7.81 (d, *J* = 8.2 Hz, 1 H), 7.49 (d, *J* = 1.6 Hz, 2 H), 7.36 (d, *J* = 7.9 Hz, 1 H), 7.16 (t, *J* = 7.6 Hz, 1 H), 7.11 (dd, *J* = 7.9, 0.9 Hz, 1 H), 4.78 (s, 2 H), 4.47 (ddt, *J* = 16.3, 8.1, 3.9, 3.9 Hz, 1 H), 4.31 (d, *J* = 13.6 Hz, 2 H), 2.99 (t, *J* = 12.3 Hz, 2 H), 2.26 - 2.39 (m, 2 H), 1.73 - 1.81 (m, 2 H).

4-(4-Bromo-2-oxo-3H-benzimidazol-1-yl)-*N*-(4-iodo-3- methyl-phenyl)piperidine-1-carboxamide (TH6943).

A mixture of 4-iodo-3-methyl-aniline (23 mg, 0.10 mmol), diisopropylethylamine (0.035 ml, 0.20 mmol), and trichloromethyl carbonochloridate (0.0060 ml, 0.050 mmol) was stirred in DCM (2.0 ml) at 20°C for 5 min. Then the mixture was added to a vial charged with 7-bromo-3-(4-piperidyl)-1H-benzimidazol-2-one 2,2,2-trifluoroacetic acid (41 mg, 0.10 mmol), diisopropylethylamine (0.017 ml, 0.10 mmol) and DCM (2.0 ml). The resulting mixture was stirred at 20°C for 3 h. The solids were then filtered off and washed with DCM, water, and then DCM again. The solid was dried at 40°C under vacuum, no further purification was done.

LCMS [M+H]^+^ 555. ^1^H NMR (400 MHz, DMSO-*d*_6_) δ ppm 11.31 (br. s., 1 H), 8.64 (s, 1 H), 7.65 (d, *J* = 8.8 Hz, 1 H), 7.51 (d, *J* = 2.5 Hz, 1 H), 7.26 (br. d, *J* = 8.1 Hz, 1 H), 7.13–7.19 (m, 3 H), 6.96 (t, *J* = 8.1 Hz, 1 H), 4.35 - 4.45 (m, 1 H), 4.24 - 4.33 (m, 2 H), 2.89–2.99 (m, 2 H), 2.32 (s, 3 H), 2.20–2.31 (m, 2 H), 1.70 - 1.79 (m, 2 H).

### X-ray crystallography

A synthetic gene encoding hOGG1 [11–327] was expressed as a cleavable N-terminally His-tagged protein in E. coli BL21(DE3) T1R pRARE2 at 18°C and purified using HisTrap HP (GE Healthcare) followed by gel filtration using HiLoad 16/60 Superdex 200 (GE Healthcare) after tag cleavage (Thrombin, GE Healthcare). Protein was stored in 20 mM MES at pH 6.0, 200 mM NaCl, 10% glycerol, 0.5 mM TCEP, at 20 mg/ml and stored at −80°C.

Samples for co-crystallization were prepared by pre-incubation of hOGG1 (18 mg/ml) with 2–4 mM of the ligands (from a stock solution of 100 mM in DMSO). Crystals of the hOGG1:TH5487 complex were obtained from a hanging-drop vapor diffusion setup against 0.12 M Alcohols, 0.1 M Buffer System 2 pH 7.5 and 48% v/v Precipitant Mix 4 (Morpheus screen (48), Molecular Dimensions, UK). A drop of 2 μl of sample was mixed with equal amount of reservoir and incubated at 16°C. Crystals grew within 24 hours and were frozen in liquid nitrogen for data collection. Diffraction data (Supplementary Table S3) were collected at station I04 of the Diamond Light Source (Didcot, UK). A complete dataset was collected from a single crystal at 100 K. Raw data images were processed and scaled with xia2 (49), DIALS (50), and Aimless (51) using the CCP4 suite 7.0 (52). Molecular replacement was performed with the coordinates of human OGG1 (PDB code 1EBM), to determine initial phases for structure solution in Phaser (53). The working models were refined using Refmac5 (54) and manually adjusted with Coot (55) Validation was performed with Molprobity (56). Figures were drawn with PyMOL (Schrödinger, LLC, New York).

The atomic coordinates and structure factors (codes 6RLW) have been deposited in the Protein Data Bank (http://wwpdb.org).

### Statistical analysis

**Figure 1.**
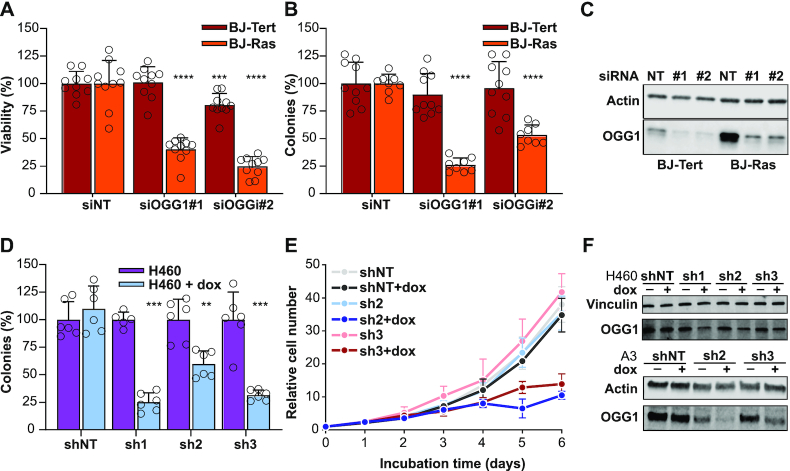
OGG1 knockdown is selectively toxic in oncogene-expressing cells and cancer cell lines. (**A**) Viability of BJ fibroblast cells immortalized with telomerase (BJ-Tert) or telomerase, SV40 large T protein and *HRAS* G12V oncogene (BJ-Ras) after OGG1 depletion for 5 days. Data are average ±SD of 10 technical replicates representative of two independent experiments. (**B**) Clonogenic survival of BJ-Tert and BJ-Ras cells following OGG1 depletion. Data are average ±SD of 8–10 replicates from three independent experiments. (**C**) Representative immunoblot analysis of OGG1 expression in BJ-Tert and BJ-Ras cells 48 h after transfection with siRNA. (**D**) Clonogenic survival of H460 cells stably transfected with doxycycline inducible shRNA constructs targeting *OGG1* (sh1–sh3) or a non-specific sequence (shNT). Cells were seeded in the presence or absence of 500 ng/ml doxycycline and colonies were enumerated after 10–12 days. Data are average ±SD of six technical replicates from two independent experiments. All values are normalized to the number of colonies in medium free of doxycycline. (**E**) Proliferation of A3 cells stably transfected with doxycycline-inducible shRNA constructs targeting *OGG1* (sh2–3) or a non-specific sequence (NT). Cells were seeded in medium with or without 250 ng/ml doxycycline and counted daily. Cultures reaching a density of more than one million cells per ml were added fresh medium to maintain cell growth and normalized to the starting density. (**F**) Representative immunoblot analysis of OGG1 expression in H460 and A3 cells stably transfected with shRNA constructs targeting *OGG1* and treated with doxycycline as in D and E. Data are average ±SD of four technical replicates from two independent experiments. Statistical significance was determined using unpaired, two-sided *t*-tests (**P < 0.01, ***P < 0.001 and ****P < 0.0001), in all cases comparing against the distribution of the corresponding non-specific RNAi-sequence.

Statistical analyses were performed with Prism 8.0 (GraphPad Software). Statistical significance Figures 1A, B, D, E, 3D, E, F, 4D, E, G, H, J, K, 5A, C, D, E, F, I, K, 6D, E, F and supplementary Figures S5A, B, S6A, S7A, B was determined using unpaired, two-sided *t*-tests (*P < 0.05, **P < 0.01, ***P < 0.001 and ****P < 0.0001) Statistical significance for Figure 3C and Supplementary Figure S4H was determined using Paired *t*-test (****P < 0.0001). Statistical significance for Supplementary Figures S1B, S1C, S4E and S4F was determined using Mann–Whitney unpaired test for nonparametric distribution (*P < 0.05, **P < 0.01, ***P < 0.001 and ****P < 0.0001). Normality of the distribution for each data set was tested using Kolmogorov–Smirnov test.

## RESULTS

### Validation of OGG1 as an anti-cancer target

While PARP inhibitors effectively block BER (16), targeting the BER pathway as such has not been validated as an anti-cancer target since PARP inhibition also has implications on replication forks (9,10), alternative end-joining (11–13) and other pathways (14). Since expression of the frequently mutated *RAS* oncogenes leads to the generation of ROS and oxidative DNA damage (19,20), we wanted to determine if OGG1, which initiates the BER process by recognizing and excising 8-oxodG, is important for the survival of transformed cells. To test this, we used siRNA to downregulate *OGG1* in the well characterized isogenic hTERT-immortalized BJ fibroblasts transformed or not with SV40 largeT antigen and *HRAS* G12V (57). Five days after transfection, we could observe a marked decrease in viability of transformed cells, but little effect in non-transformed cells (Figure 1A). Moreover, *OGG1* knockdown decreased the colony-forming ability of BJ-Ras, but not BJ-Tert cells, indicating that OGG1 protected cells against oncogene-induced stress (Figure 1B and C).

To further investigate if OGG1 depletion inhibited growth in human transformed and cancer cell lines we tested the colony forming ability in previously validated OGG1 CRISPR-Cas9 depleted epithelial kidney embryonic cells (HEK293T(KO)) (39). Whereas the number of colonies was similar in HEK293T (KO) cells compared to the parental HEK293T OGG1 proficient cells, the colony area generated by the HEK293T (KO) cells was significantly smaller (Supplementary Figure S1A, S1B and S1C). In human cancer cell lines, depletion of *OGG1* in H460 lung cancer cells stably transfected with doxycycline-inducible small hairpin RNA (shRNA) constructs targeting *OGG1* reduced clonogenic ability (Figure 1D and F). A3 T-cell acute lymphoblastic leukemia cells transduced with the same constructs divided normally for 48 h after addition of doxycycline followed by slower proliferation and a loss of viability thereafter (Figure 1E and F).

**Figure 2.**
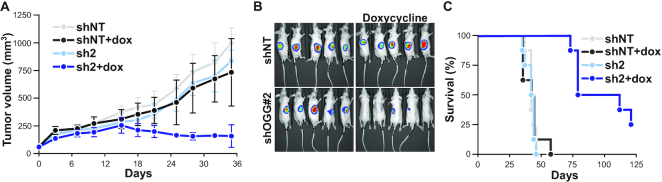
OGG1 knockdown reduces tumor growth *in vivo*. (**A**) A3 cells harbouring luciferase and a doxycycline-inducible shRNA construct targeting *OGG1* or a non-specific sequence were injected subcutaneously in mice. Doxycycline was added to the drinking water at day 7, and tumor growth were monitored twice a week thereafter. Data are average ± SD, *n* = 8 per group. (**B**) Bioluminescence of luciferase expressing A3 cells in five representative mice imaged 28 days after grafting. (**C**) Survival of animals grafted with A3 cells. Mice were euthanized when tumor size reached 1000 mm^3^.

We then stably expressed luciferase in the same A3 inducible cell line, which was injected subcutaneously into Balb/C nude mice. Doxycycline was added to the drinking water when tumor volume had reached 200 mm^3^. This caused a regression in tumor size in xenografts harboring *OGG1*-targeting shRNA, whilst tumors with non-targeting shRNA were unaffected (Figure 2A and B, Supplementary Figure S2). After seven weeks, only mice injected with *OGG1*-knockdown cells remained alive (Figure 2C). These data show that OGG1 protects cancer cells from oncogenic stress *in vivo* and validate OGG1 as anti-cancer target.

### OGG1 active site inhibitors suppress cancer cell growth

**Figure 3.**
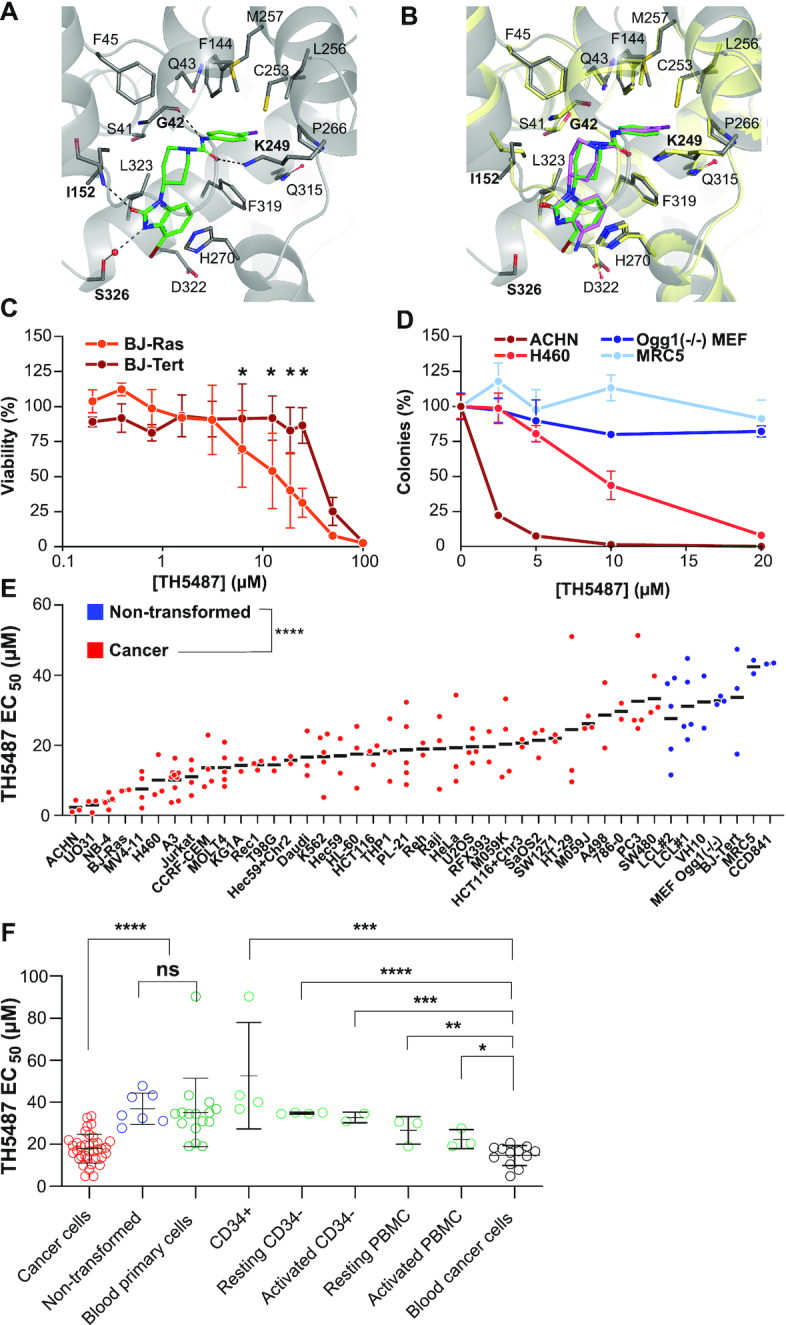
The OGG1 inhibitor TH5487 is selectively toxic to oncogene-expressing cells and cancer cell lines. (**A**) Close-up view of ligand TH5487 (green) binding to human OGG1. Important residues in the binding site are marked, hydrogen bond interactions are shown in black dashed line, water (red sphere)-mediated interactions in grey dashed line. (**B**) Comparison between the binding of ligand TH5487 (green) to human OGG1 (gray) with the structure of TH5675 (pink) bound to mouse OGG1 (yellow, PDB 6G3Y). (**C**) Viability of BJ-Tert and BJ-Ras cells exposed to the indicated concentrations TH5487 for five days. Data are average ±SD of four technical replicates from two independent experiments. (**D**) Clonogenic survival of cell lines exposed to TH5487. The cancer cell lines ACHN and H460, and the non-transformed cell lines MRC5 and *Ogg1*^−/−^ mouse embryonic fibroblasts (MEF) were incubated for 6–11 days in the presence of the indicated concentrations TH5487, followed by colony enumeration. Data are average ±SD values of four technical replicates, representative of three independent experiments. (**E**) TH5487 selectively decreases viability of cancer cell lines. EC50-values of 34 cancer (red) and 7 non-transformed cell lines (blue). Cells were exposed to a dilution series of TH5487 for five days followed by a viability assessment using resazurin. All cell lines were tested in two to ten independent experiments. Each point represents the EC50-value from one experiment (average of two or three technical replicates). (**F**) Comparative analysis of EC_50_ values for TH5487 in cancer- and non-transformed cell lines, and CD34+ hematopoietic stem cells, CD34- fraction from cord blood or PBMCs from healthy donor blood. All primary blood cells were tested upon activation with Dynabeads/Phytohemagglutinin or not and exposed to a dilution series of TH5487 to calculate EC_50_ values by resazurin assay. Data for blood cells are average ±SD from two to four independent donors, and are significantly different from the hematological cancer cell lines tested, using a two-sided unpaired *t*-test (*P < 0.05, **P < 0.01, ***P < 0.001 and ****P < 0.0001, ns, non-significant).

We previously described TH5487 as a selective small molecule inhibitor of OGG1 (39). To further understand the precise function of this inhibitor, we here determined the X-ray crystal structure of human OGG1 in complex with TH5487 (Figure 3A), showing that it binds in the active site, in an opposite orientation from the natural substrate (39), with the iodophenyl tail of TH5487 occupying the place of the 8-oxoguanine base. The OGG1 protein undergoes a conformational change when binding DNA, into a closed structural form. Here, we observe the benzimidazolone core interacts with a lipophilic exo-site outside the active site, stabilized by Ile152 and Leu323 in addition to a π-stacking interaction with His270. Also, the carbonyl side group of TH5487 makes a hydrogen bond with the Ile152 α-amino backbone. Finally, the bromine atom of TH5487 makes a water-mediated interaction with the side chain of Ser326. This confirms the overall molecular mechanism of this class of inhibitors (Figure 3B and Supplementary Figure S3), where OGG1 adopts a closed conformation upon binding TH5487, thereby blocking OGG1 from accessing substrate DNA lesions in DNA and chromatin (39).

To study if TH5487 selectively inhibited growth of transformed cells, we incubated the pair of isogenic BJ-fibroblasts with the inhibitor. TH5487 causes a concentration-dependent decrease in viability in BJ-Ras fibroblasts (Figure 3C), in line with reduced survival after RNAi-mediated *OGG1* downregulation (Figure 1A). Furthermore, the isogenic hTERT-immortalized cells are less sensitive to TH5487 than BJ-Ras (EC50 = 29.5 ± 4.7 μM and EC50 = 11.4 ± 5.7 μM, respectively, *P* < 0.05) (Figure 3C), in line with high tolerance to *OGG1* siRNA (Figure 1A). We then characterized a subset of adherent cell lines in colony formation assays and find TH5487 causes a concentration-dependent loss of clonogenic potential in the cancer cell lines ACHN and H460, whereas the non-transformed cell lines MRC5 and *Ogg1*^−/−^ mouse embryonic fibroblasts were unaffected by treatment up to 20 μM (Figure 3D). These results suggest that TH5487 may selectively kill cancer cells. To test this more broadly, we determined the sensitivity of a large panel of cancer and non-transformed cell lines and found overall that TH5487 caused loss of viability in cancer cells while being better tolerated in non-transformed cell lines (Figure 3E and Supplementary Table S1). Moreover, this selectivity was not unique to TH5487, since the TH5487 inhibitor analogues TH5796 and TH6943 displayed a similar growth inhibition specific to cancer cells (Supplementary Figure S4A to S4F and Supplementary Table S1).

Finally, TH5487 was well tolerated by a set of hematopoietic primary cells derived from healthy donors (CD34+ cells, resting and activated CD34- cells from umbilical cord blood (Figure 3F). Taken together, TH5487 caused loss of proliferation to a higher degree in the tested cancer cell lines (EC50 17.9 ±6.9 μM) compared to non-transformed cell lines (EC50 36.9 ± 7.5 μM) (Figure 3F). Hematological cancer cell lines were particularly sensitive compared to primary blood cells (EC50 14.7 ± 4.7 μM and 35.1 ± 20 μM, *P* = 0.00029).

### Anticancer properties overlap between OGG1 loss and OGG1 inhibitors

**Figure 4.**
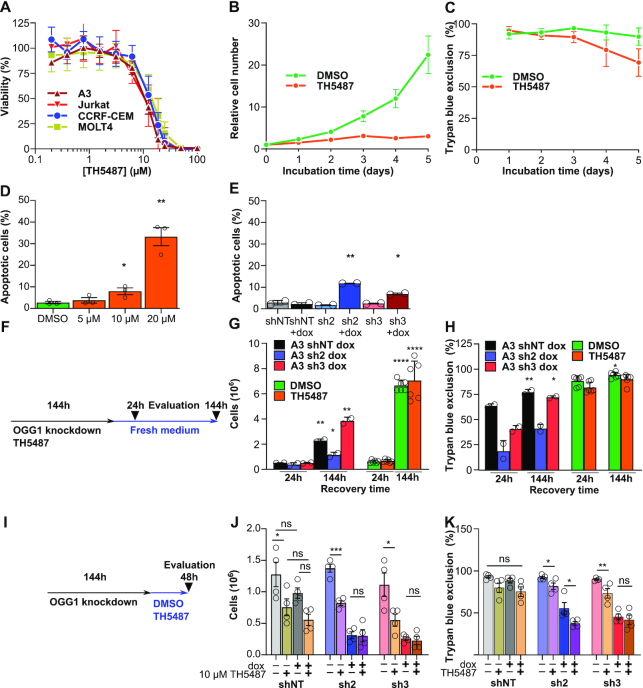
Anticancer properties overlap between OGG1 loss and OGG1 inhibitors. (**A**) Viability of T-cell acute leukoblastic leukemia cell lines treated for 5 days with the indicated doses TH5487. Data are average ±SD of independent experiments (A3, *n* = 5; Jurkat, *n* = 3; MOLT-4, *n* = 4; CCRF-CEM, *n* = 3). (**B**) Relative cell numbers and (**C**) viability (%) for A3 cells treated for 5 days with 10 μM TH5487. Data are average ±SD of six replicates from three independent experiments. (**D**) Induction of apoptosis in A3 cells treated for 72 h TH5487 and stained for Annexin V. Data are average ±SD of three independent experiments. (**E**) A3 cells transfected with shRNA targeting OGG1 were treated with doxycycline for 6 days and stained for Annexin V. Data are average ±SD of two independent experiments. (**F**) Scheme for recovery experiment in which A3 *OGG1*-shRNA cells or A3 cells were silenced or inhibited for 6 days with doxycycline (200 nM) or TH5487 (10 μM). Then, 250 000 cells/ml were seeded in fresh media and recovered for 24 h and 144 h by measuring cell number (**G**) and viability (**H**). Data are average ±SD of 2–6 technical replicates representative of two independent experiments (**I**). Scheme for off-target effect evaluation in which A3 *OGG1*-shRNA cells were exposed to doxycycline for six days and after that, cells were exposed to TH5487 (10 μM) for 48 h followed by measurements of cell numbers (**J**) and viability by trypan blue exclusion (**K**). Data are average ±SD of two technical replicates from four independent experiments. Statistical significance was determined using unpaired, two-sided *t*-tests (*P < 0.05, **P < 0.01, ***P < 0.001, ****P < 0.0001, ns, non-significant).

We selected A3 cells as a model cell line to characterize anti-proliferative properties mediated by either OGG1 inhibition by TH5487 or by shRNA knockdown of *OGG1*. We found that similar to *OGG1* knockdown (Figure 1E), TH5487 inhibited proliferation of A3 and other T-cell acute leukoblastic leukemia cell lines with an EC_50_ of ∼10 μM (Figure 4A and Supplementary Table S1). A3 cells exposed to 10 μM TH5487 showed slower proliferation for up to 72 h (Figure 4B), followed by a mild decrease in cell viability (Figure 4C). The same dose of TH5487 was well tolerated by resting and activated peripheral blood mononuclear cells (PBMCs) and primary T-cells from healthy donors (Supplementary Figure S4G to S4K). Using Annexin V-propidium iodide staining, we could demonstrate a slight but significant increase in apoptosis after OGG1 knockdown and 5 and 10 μM TH5487 treatment, which was more substantial at 20 μM (Figure 4D and E). To further characterize the cell proliferation defect, we released the cells into fresh medium after treatment. Similar to A3 *OGG1* knockdown cells, A3 cells exposed for 6 days to 10 μM TH5487 were able to divide normally after replacing with fresh cell culture media (Figure 4F to H) showing proliferation defects due to OGG1 depletion or targeting with TH5487 to be reversible. This suggests that TH5487 might primarily cause cytostatic rather than cytotoxic effects.

To test whether the antiproliferative effect of TH5487 was related to OGG1 inhibition, *OGG1* knockdown cells treated with doxycycline for 5 days were challenged for an additional 48 h with 10 μM TH5487. However, no significant additional toxic effect was detected (Figure 4I–K). Alternatively, A3 *OGG1* shRNA cells were exposed to doxycycline alone or in combination to TH5487 for 6 days and we found that TH5487 had additional toxic effect for one of the constructs (A3 sh2) but not for the other (A3 sh3) (Supplementary Figure S5A and S5B). Interestingly, the combination of TH5487 and doxycycline in A3 sh2 knockdown cells led to a partial recovery of OGG1 protein levels, which might explain why TH5487 has an additional effect for this specific construct (Supplementary Figure S5C and S5D). Finally, to further investigate potential off-target effects of the compound in an OGG1 knockout model, we evaluated the clonogenic potential of HEK293T or HEK293T(KO) cells exposed to TH5487 for a period of 8 days. Here, we found that the colony area was significantly reduced in the parental HEK293T OGG1 proficient cells compared to HEK293T (KO) cells (Supplementary Figure S5E and S5F). These results suggest that the antiproliferative properties of TH5487 in cancer cells are primarily related to OGG1 inhibition although residual off target effects at higher concentrations (20 μM or more) cannot be excluded, since it is a common mechanism of action of cancer drugs (58).

### Targeting OGG1 results in replication stress

**Figure 5.**
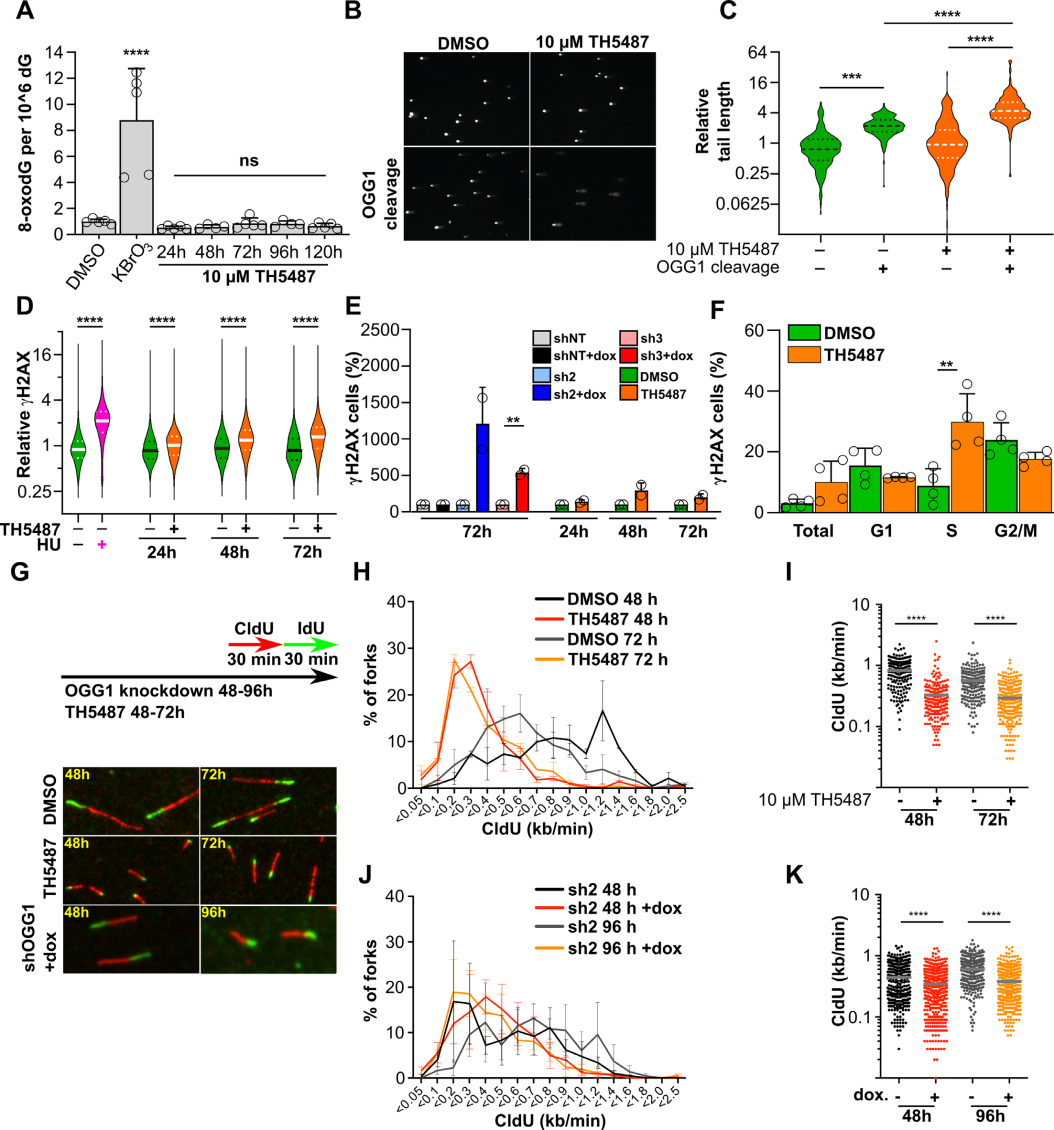
Effect of TH5487 on DNA lesions, DNA damage markers and DNA replication. (**A**) 8-oxodG accumulation in A3 cells. A3 cells were treated with 20 mM KBrO_3_ for 1 h or with 10 μM TH5487 for the indicated times, and the amount of genomic 8-oxodG was quantified with LC-MS/MS. Data are average ±SD of five replicates from two independent experiments. (**B**) Comet assay. A3 cells were treated with 10 μM TH5487 for 72 h and strand-breaks and OGG1 substrate lesions were analyzed with the OGG1-modified Comet assay. Representative images of cells are shown. (**C**) Violin blot of Comet tail moment. Cells were treated as in B, and the tail moment of the cells were analyzed using blinded automatic analysis (*n* = 200 per condition from two independent experiments). The full line indicates median, and the dotted lines quartiles. (**D**) Violin blot of phosphorylated γH2AX intensity. A3 cells were treated with 0.1% DMSO or 10 μM TH5487 for the indicated times and stained for phosphorylated γH2AX. Treatment with 2 mM Hydroxyurea for 1 h was used as positive control. At least 33 000 nuclei per group were quantified from three independent experiments. The full line indicates median, and the dotted lines quartiles. All values are normalized to that of the mean value of the 1 h non-treated sample. (**E**) Relative induction of positive γH2AX cells in *OGG1* shRNA depleted cells for 72 h or OGG1 inhibited with 10 μM of TH5487 for 24, 48 and 72 h. Data are average ±SD of two technical replicates representative of 1–2 independent experiments (**F**) Relative cell cycle distribution of positive γH2AX gated cells along G1, S or G2/M cell cycle phases after 72 h treatment with 10 μM TH5487. Data are average ±SD of two technical replicates from two independent experiments. (**G**) Experimental setup of DNA fiber assay. A3 cells were treated with 0.1% DMSO or 10 μM TH5487 for 48 h or 72 h, alternatively A3 shOGG#2 were treated with doxycycline for 48 or 96 h followed by addition of 5-chloro-2′-deoxyuridine (CldU) or 5-iodo-2′-deoxyuridine (IdU) to the medium. Representative images of DNA replication fibers are shown. (**H**) Distribution of fork speed in CldU-labelled A3 cells treated with DMSO or TH5487 for 48 or 72 h. (**I**) Total fork speed in DMSO and TH5487-treated cells. (**J**) Distribution of fork speed in CldU-labelled A3 shOGG1#2 cells treated or not with doxycycline for 48 or 96 h. (**K**) Total fork speed in A3 shOGG1#2 cells treated or not with doxycycline. Data shown as average ± SD from three independent experiments. At least 300 forks were scored per condition. Statistical significance was determined using unpaired, two-sided *t*-tests (**P < 0.01, ***P < 0.001 and ****P < 0.0001; ns, non-significant).

Given the general high levels of oncogene-induced ROS and associated oxidative DNA damage in cancer (18–23), we were curious if the loss of viability was preceded by genomic accumulation of oxidized DNA. To test this, we exposed A3 cells to 10 μM TH5487 for up to five days, purified genomic DNA, and measured the genomic content of 8-oxodG. Unexpectedly, 8-oxodG levels did not rise above assay background levels during the experiment (Figure 5A). Nor did 8-oxodG rise above background in OGG1-depleted A3 cells (Supplementary Figure S6A). Using a more sensitive method, we could detect genomic OGG1 substrates in A3 cells using the modified Comet assay. Treatment with 10 μM TH5487 caused a ∼50% increase in OGG1-induced tail length, indicating that TH5487 induced a small increase in OGG1 substrate levels in these cells (Figure 5B and C). The overall increase in nuclear 8-oxodG in DNA is minor in comparison to KBrO_3_ control and unlikely to explain growth inhibition induced by OGG1 inhibitors or protein loss.

In spite of few nuclear oxidative DNA lesions, we found unexpectedly a significant increase in phosphorylated γH2AX, detected after 24 h treatment with 10 μM TH5487 as well as following shRNA depletion of *OGG1* (Figure 5D and E). Interestingly, the distribution of γH2AX positive cells after treatment by OGG1 inhibitors during cell cycle revealed that the DNA damage induced by TH5487 was confined to S-phase cells (Figure 5F), indicating that OGG1 deficiency may affect DNA replication. However, the A3 cell cycle distribution after OGG1 perturbation remained largely constant, although we observed an accumulation in the sub-G1 population (Supplementary Figure S6B and S6C). Yet, OGG1 inhibition induced a marked reduction in replication fork rate at both 48- and 72 h time points (Figure 5G to I), largely mimicked by *OGG1* depletion by RNAi at both 48- and 96-h time points (Figure 5G, J and K). Furthermore, experiments where cells were pulsed with 5-ethynyl-2′-deoxyuridine (EdU) followed by click-chemistry mediated detection of newly incorporated nucleotides, supported that DNA synthesis was reduced in OGG1 inhibited and *OGG1*-depleted cells (Supplementary Figure S7). Together, these data demonstrate that the replicative potential of OGG1 perturbed cells may be caused by replication stress in the form of a lowering of replication fork rate and DNA damage during S-phase.

**Figure 6.**
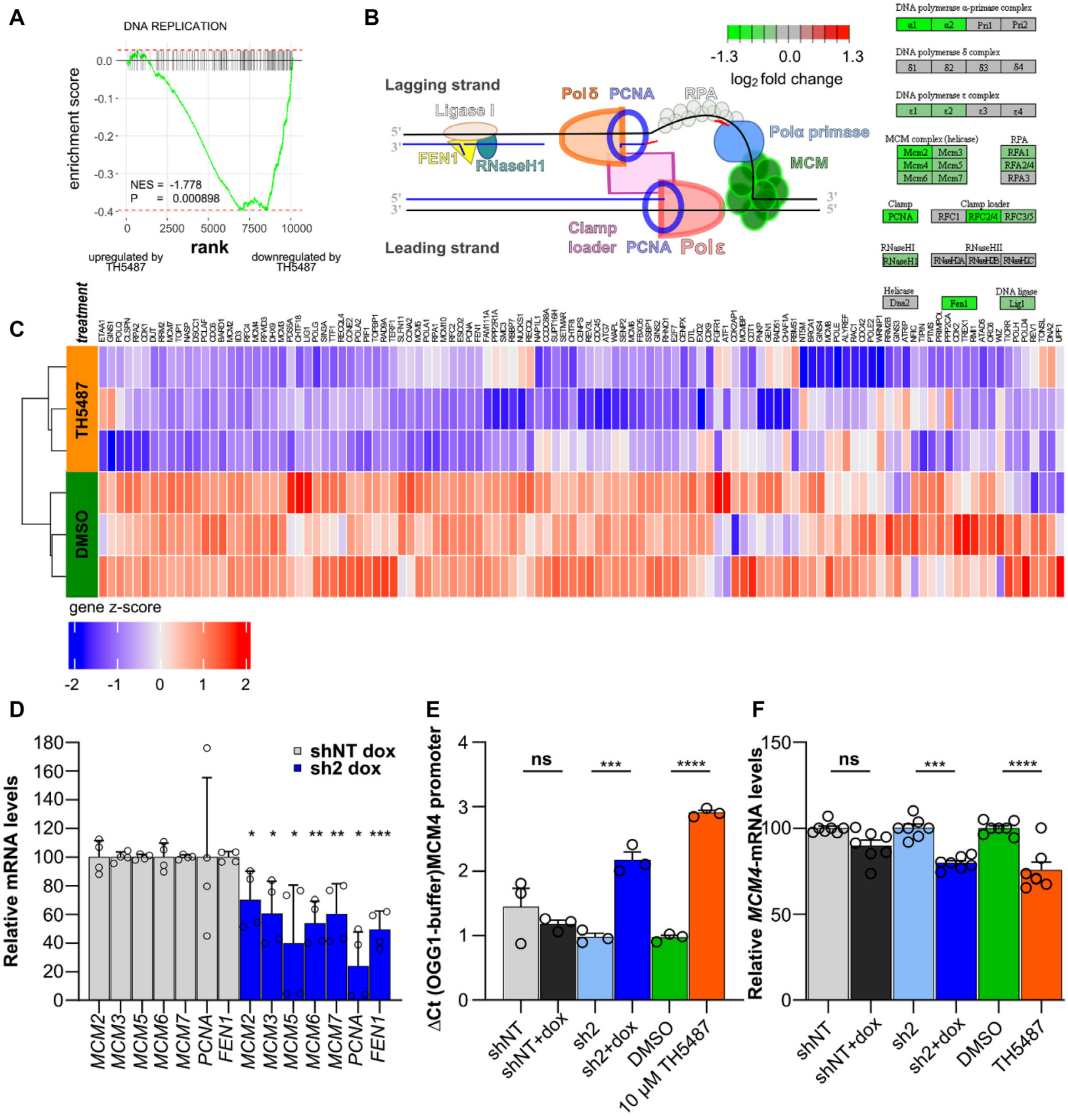
TH5487 treatment induces a downregulation of DNA replication genes (**A**) Gene Set Enrichment analysis plot of the DNA Replication Gene Ontology Gene Set. A3 shNT cells were treated with 10 μM TH5487 for 24 h or DMSO as control, and RNA sequencing was performed. Genes with a mean normalized count > 5 were ranked by log2 fold change. Genes towards the top of the ranked gene list are upregulated after TH5487 treatment, genes with higher ranks are downregulated. NES: normalized enrichment score, P value has been adjusted for multiple testing using the Benjamini–Hochberg method. (**B**) Log_2_ fold changes in expression level of DNA Repair genes of TH5487 treated A3 control cells compared to DMSO treated cells. Only genes with a mean normalized count >5 were analyzed. Visualization of the KEGG DNA Replication pathway. (**C**) Heatmap of the leading edge genes from the DNA Replication Gene Ontology Gene Set. Genes and samples were unsupervisedly clustered using hierarchical clustering (euclidean distance, complete linkage). Rows indicate technical replicates (n = 3). A negative *z*-score indicates relative downregulation, a positive relative upregulation of the gene. (**D**) Relative mRNA expression levels of replication-associated genes in *OGG1*-depleted cells (normalized to A3 shNT and expressed in percentage). A3 shNT or sh2-cells were treated with doxycycline for 72 h. Data shown as average ± SEM from four technical replicates from two independent experiments. (**E**) Oxidation at *MCM4* promoter. The indicated cells were treated with 10 μM TH5487 or 250 ng/ml doxycycline for 72 and 96 h, respectively, DNA was purified and treated or not with OGG1 followed by PCR amplification of the *MCM4* promoter. Data shown are the difference in Ct values caused by OGG1 enzymatic treatment or buffer. Data represent average ± SEM of three technical replicates from two independent experiments. (**F**) Relative expression of *MCM4* mRNA levels in OGG1 perturbed cells expressed in percentage. Cells were treated as in D. and data represent average ± SEM 3–4 technical replicates from two independent experiments. Statistical significance was determined using a two-sided unpaired *t*-test (ns, non-significant, *P < 0.05, **P < 0.01, ***P < 0.001 and ****P < 0.0001).

To explore how OGG1 inhibition causes replication stress we performed RNA sequencing with A3 cells treated with 10 μM TH5487 or DMSO for 24 h. Gene Set Enrichment Analysis (GSEA) of the differentially expressed genes revealed that ‘DNA replication’ was one of the most downregulated gene signatures (Figure 6A and Supplementary Figure S8). Genes encoding key proteins and enzymes necessary for DNA replication were downregulated upon TH5487 treatment (Figure 6B and C). We validated a panel of replication associated genes following OGG1 knockdown, confirming a ∼50% downregulation after 96 h for all eight investigated transcripts (Figure 6D). These results suggest that TH5487 and OGG1 knockdown induce early alterations in the A3 transcriptional profile that include downregulation in DNA replication signature. These are unlikely to be explained by a lack of replicating cells, since cells in S-phase were not depleted from the cell population in any experimental condition (Supplementary Figure S6B and S6C).

Interestingly, the promoter sequence of several genes included in the ‘DNA replication’ gene set contains one or several SP1 transcription factor binding motifs (https://epd.epfl.ch//index.php). Since OGG1 has been reported to be required for recruitment of transcription factors such as SP1 to the promoter of genes containing motifs of recognition for this transcription factor (59), we exposed A3 cells or *OGG1*-shRNA A3 cells to TH5487 for 72 h or doxycycline for 96 h, respectively, and assessed promoter oxidation at the SP1 binding motif contained within the *MCM4* promoter. We observed a significant accumulation of oxidative DNA damage at this position that correlated with a slight but significant downregulation of *MCM4* mRNA levels (Figure 6E and F).

**Figure 7.**
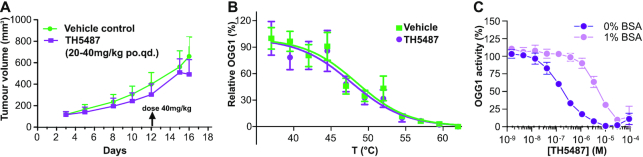
OGG1 inhibitors does not inhibit tumor growth *in vivo*. (**A**) Lack of TH5487 efficacy *in vivo*. A3 cells were injected subcutaneously in mice, allowed to graft for three days and then administered 20–40 mg/kg TH5487 orally, once a day on weekdays. Twelve days into the treatment, the dose was increased to 40 mg/kg. Data are average ± SD (*n* = 9 per treatment group). (**B**) TH5487 target engagement in A3 xenograft. Tumors were excised following termination, and TH5487 target engagement was analyzed by cellular thermal shift assay, using an antibody specific for the human OGG1 protein. Data are average ± SD (*n* = 4 different tumors per group). (**C**) Effect of bovine serum albumin on OGG1 inhibition by TH5487. A dilution series of TH5487 was incubated with OGG1 enzyme and a DNA reporter oligonucleotide containing an OGG1-substrate, in the presence or absence of 1% (w/v) bovine serum albumin. Data are average ± SD of four technical replicates from two independent experiments.

Since our data indicated that OGG1 inhibitor treatment slowed A3 cell proliferation, we wanted to test if treatment with TH5487 could suppress growth of A3 xenograft tumors in mice. We orally administered 20 mg/kg TH5487 in established subcutaneous tumors of A3 cells. However, we were not able to detect xenograft growth inhibition, even after an increase in dose to 40 mg/kg after the first week (Figure 7A). Since we were unable to demonstrate OGG1 target engagement in A3 cells in the derived xenograft tumor (Figure 7B), we hypothesized that the lack of *in vivo* TH5487 efficacy was due to lack of OGG1 target engagement.

As TH5487 was shown to be stable in mouse serum (39), we investigated if TH5487 interacted with serum proteins. Thus, we incubated bovine serum albumin proteins together with TH5487 and observed that bovine serum albumin induced a marked loss of inhibition of OGG1 enzyme activity (Figure 7C). This suggests that albumin competed with OGG1 enzyme for TH5487 and severely reduced its efficacy. Thus, we conclude that systemic administration of TH5487 is not feasible with the current molecule and new compounds or formulation strategies have to be developed for effective treatment of cancer.

## DISCUSSION

Despite the inherent high levels of ROS and DNA damage in cancer, and the clinical success of BER pathway inhibitors, DNA repair pathways removing oxidative DNA damage have so far not been pharmacologically targeted (35). We and others (39,60,61) recently reported the first examples of cell active inhibitors targeting OGG1, and here we provide the first structural details of how TH5487 binds to the human enzyme (Figure 3A and B). Importantly, we show that multiple cancer cell lines are sensitive to loss or inhibition of OGG1, which could relate to an underlying addiction to a functional OGG1 in cancer. This may in part explain why *Ogg1* knockout mice are largely spared from cancer (62), and the relative rarity of the expected C→A mutation signature in human cancers (26) in spite of the well-demonstrated fundamental role of ROS in hyperplasias. Surprisingly, after treatment with OGG1 inhibitor the absolute levels of genomic 8-oxodG remained low, at or above background levels of one 8-oxodG per million guanines (corresponding to about 3000 damaged bases per diploid genome), even after prolonged treatments with TH5487. This finding suggests that a sizeable number of 8-oxodG in bulk chromosomal DNA of cancer cells must be very low, even in stressed cells. This may have broad implications for 8-oxodG as a biomarker in cancer research since it questions the usefulness and applicability of indirect detection of genomic 8-oxodG in whole cells, using single-cell electrophoresis and antibody- or avidin-staining. Instead, novel methods to map and study specific genomic locations enriched with 8-oxodG lesions have been developed. For example, mapping of 8-oxodG in mouse embryonic fibroblasts (MEF) and MCF10A breast epithelial cells, has revealed that 8-oxodG is enriched at promoters and 5′ and 3′ untranslated regions rather than randomly distributed (63–65). In MEF cells, ∼10 000 regions of 8-oxodG were enriched in WT mouse embryonic fibroblasts compared to ∼18 000 regions when *Ogg1* was knocked out (63). Interestingly, 21 out of the 113 replication genes transcriptionally downregulated by OGG1 inhibitors (*ATG, CDK1, CDK2, CDK2AP1, DNA2, E2F7, FEN1, MCM3, MCM4, NAP1L1, NFIC, NUCKS1, PDS5A, POLD4, RAC1, RAD51, RAD9A, RFC4, SENP2, SIN3A, SMC3*), were included in the list of chromosomal gene locations having 8-oxodG-enriched peaks in *Ogg1*^−/−^-MEF (63). This observation, together with our data showing that OGG1 inhibition/depletion induces accumulation of oxidative DNA damage at *MCM4* gene promoter, and downregulation of *MCM4* mRNA levels, supports that OGG1 might play a role in 8-oxodG epigenetic regulation of gene expression. This adds to the growing list of observations where DNA oxidation in promoter sequences modulates gene transcription.

Indeed, our transcriptional study to evaluate the impact of TH5487 in A3 cells revealed that differential gene expression are observed 24 h after OGG1 inhibition. OGG1 inhibition induced transcriptional downregulation of genes involved in different biological processes such as cell cycle control and DNA replication (Figure 6 and Supplementary Figure S8). This occurred earlier compared to some of the replication stress phenotypes associated with OGG1 inhibition/downregulation observed in other assays. For example, reduction in EdU incorporation was only detectable after 96 h treatment with TH5487 or doxycycline in A3 cells or *OGG1*-shRNA A3 cells respectively (Supplementary Figure S7). In addition, we have found reduced transcriptional levels for all genes encoding for MCM2–7 complex after TH5487 treatment (Figure 6) followed by a progressive accumulation of γH2AX positive A3 cells during S-phase at 72 h (Figure 5). These results are not surprising considering that MCM proteins are required for processive DNA replication and that loss of MCM function causes DNA damage and genome instability (66,67). This suggests that transcriptional downregulation of DNA replication genes might be the earliest phenotype associated with TH5487 induced replication stress in A3 cells.

Taking into consideration the selectivity of TH5487 for arresting cancer cell proliferation (Figure 3), and that amplification and/or upregulation MCMs members have been identified as biomarkers of progression and negative outcome in several cancer types (68), including OGG1 inhibition in clinical prevention strategies for cancer treatment, based on the MCMs expression status, could be of benefit. On the other hand, the ready reversibility of TH5487 in cell proliferation supports the cytostatic nature of OGG1 inhibitors (Figure 4). Since cancer intervention using cytostatic drugs alone is often ineffective, combination strategies using OGG1 inhibitors together with other cancer-selective or cytotoxic drugs should be rationalized and tested in preclinical cancer models to address the utility of OGG1 inhibitors for cancer treatment. For that, significant improvements to its formulation may be required for the compound to show *in vivo* efficacy in longer term experiments such as cancer xenograft studies. In particular, the affinity to albumin reported here, which cause more than 99% of the molecule to associate with plasma proteins (39) is a challenge that must be solved for TH5487 to be useful for *in vivo* applications where OGG1 must be targeted over a long period of time to induce cell death and inhibit proliferation. Alternatively, differences between mouse strains, formulation or mode of administration may account for the different outcomes of *in vivo* experiments involving TH5487. Moreover, although TH5487 is well tolerated by mice (39), the possibility for off-target effects at high doses (see e.g. Figure 4D) must be taken into account.

In conclusion, we validate OGG1 as one more to the list of existing BER factors, e.g., PARP1, as potential targets for treatment of cancer.

## DATA AVAILABILITY

The atomic coordinates and structure factors (codes 6RLW) have been deposited in the Protein Data Bank (http://wwpdb.org). Transcriptome data is deposited at the Gene Expression Omnibus with the accession number GSE155782. The data that support the findings of this study are available from the corresponding author upon reasonable request.

## Supplementary Material

gkaa1048_Supplemental_File
