# Supplementary material for: Targeting OGG1 arrests cancer cell proliferation by inducing replication stress
Source: Nucleic Acids Res. 2020 Nov 19;48(21):12234–51. doi: 10.1093/nar/gkaa1048 (PMC7708037; doi:10.1093/nar/gkaa1048)
Supplement: gkaa1048_Supplemental_File [file gkaa1048_supplemental_file.pdf]

SUPPLEMENTARY MATERIALS

Targeting OGG1 arrests cancer cell proliferation by inducing replication stress

Torkild Visnes[1,2,*,†], Carlos Benítez-Buelga[1,†], Armando Cázares-Körner[1], Kumar Sanjiv[1], Bishoy M F Hanna[1], Oliver Mortusewicz[1], Varshni Rajagopal[1], Julian J Albers[1], Daniel W Hagey[3], Tove Bekkhus[1], Saeed Eshtad[1], Juan Miguel Baquero[4], Geoffrey Masuyer[5,6], Olov Wallner[1], Sarah Müller[1], Therese Pham[1], Camilla Göktürk[1], Azita Rasti[1], Sharda Suman[1], Raúl Torres-Ruiz[7,8], Antonio Sarno[9,10,11], Elisée Wiita[1], Evert J Homan[1], Stella Karsten[1], Karthick Marimuthu[1], Maurice Michel[1], Tobias Koolmeister[1], Martin Scobie[1], Olga Loseva[1], Ingrid Almlöf[1], Judith Edda Unterlass[1], Aleksandra Pettke[1], Johan Boström[1], Monica Pandey[12], Helge Gad[12], Patrick Herr[12], Ann-Sofie Jemth[1], Samir El Andaloussi[3], Christina Kalderén[1], Sandra Rodriguez-Perales[6], Javier Benítez[3,13], Hans E Krokan[9,10], Mikael Altun[1], Pål Stenmark[3,14], Ulrika Warpman Berglund[1], Thomas Helleday[1,12,*]

Affiliations

1) Science for Life Laboratory, Department of Oncology-Pathology, Karolinska Institutet, S-171 76 Stockholm, Sweden.

2) Department Biotechnology and Nanomedicine, SINTEF Industry, N-7465 Trondheim, Norway.

3) Department of Laboratory Medicine, Karolinska Institutet, Stockholm, Sweden.

4) Human Genetics Group, Spanish National Cancer Research Centre (CNIO), Madrid, Spain.

5) Department of Biochemistry and Biophysics, Stockholm University, SE-106 91 Stockholm, Sweden

6) Department of Pharmacy and Pharmacology, Centre for Therapeutic Innovation, University of Bath, Bath BA2 7AY, UK

7) Molecular Cytogenetics Group, Human Cancer Genetics Program, Spanish National Cancer Research Centre (CNIO), Madrid, 28029, Spain.

8) Josep Carreras Leukemia Research Institute and Department of Biomedicine, School of Medicine, University of Barcelona, Barcelona 08036, Spain.

9) Department of Clinical and Molecular Medicine, Norwegian University of Science and Technology, Trondheim, Norway.

10) The Liaison Committee for Education, Research and Innovation in Central Norway, Trondheim, Norway.
11) Department of Environment and New Resources, SINTEF Ocean, N-7010 Trondheim, Norway.

12) Weston Park Cancer Centre, Department of Oncology and Metabolism, University of Sheffield, Sheffield S10 2RX, UK.

13) Spanish Network on Rare Diseases (CIBERER), Madrid, Spain.

14) Department of Experimental Medical Science, Lund University, SE-221 00 Lund Sweden

* Corresponding authors T.V. torkild.visnes@sintef.no, T.H. t.helleday@sheffield.co.uk
† Contributed equally

Supplementary Figures

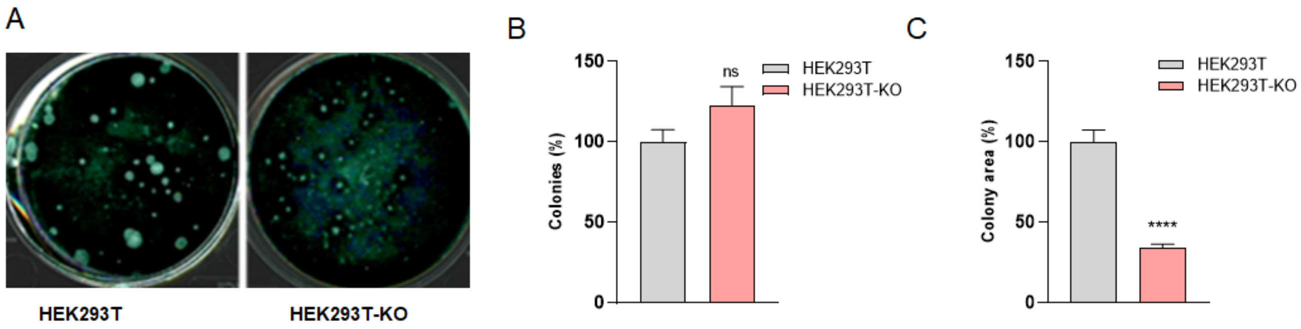

Supplementary Fig. S1: Colony size is decreased in OGG1-knockout cells. A. Colour contrasted HEK293T or HEK293T(KO) colonies from scanned 6 well plates. Colonies were grown for 8 days from seeding. **B.** Colonies (%) relative to parental HEK293T. **C.** Colony area measured in pixels for OGG1 proficient HEK293T or CRISPR-Cas9 *OGG1* knockout (KO). Data are average ±SEM of 4 technical replicates from 2 independent experiments. Statistical significance was determined using Mann Witney test (ns, non-significant; ****, P<0.0001), in all cases comparing against the distribution of the corresponding wild-type cell line.

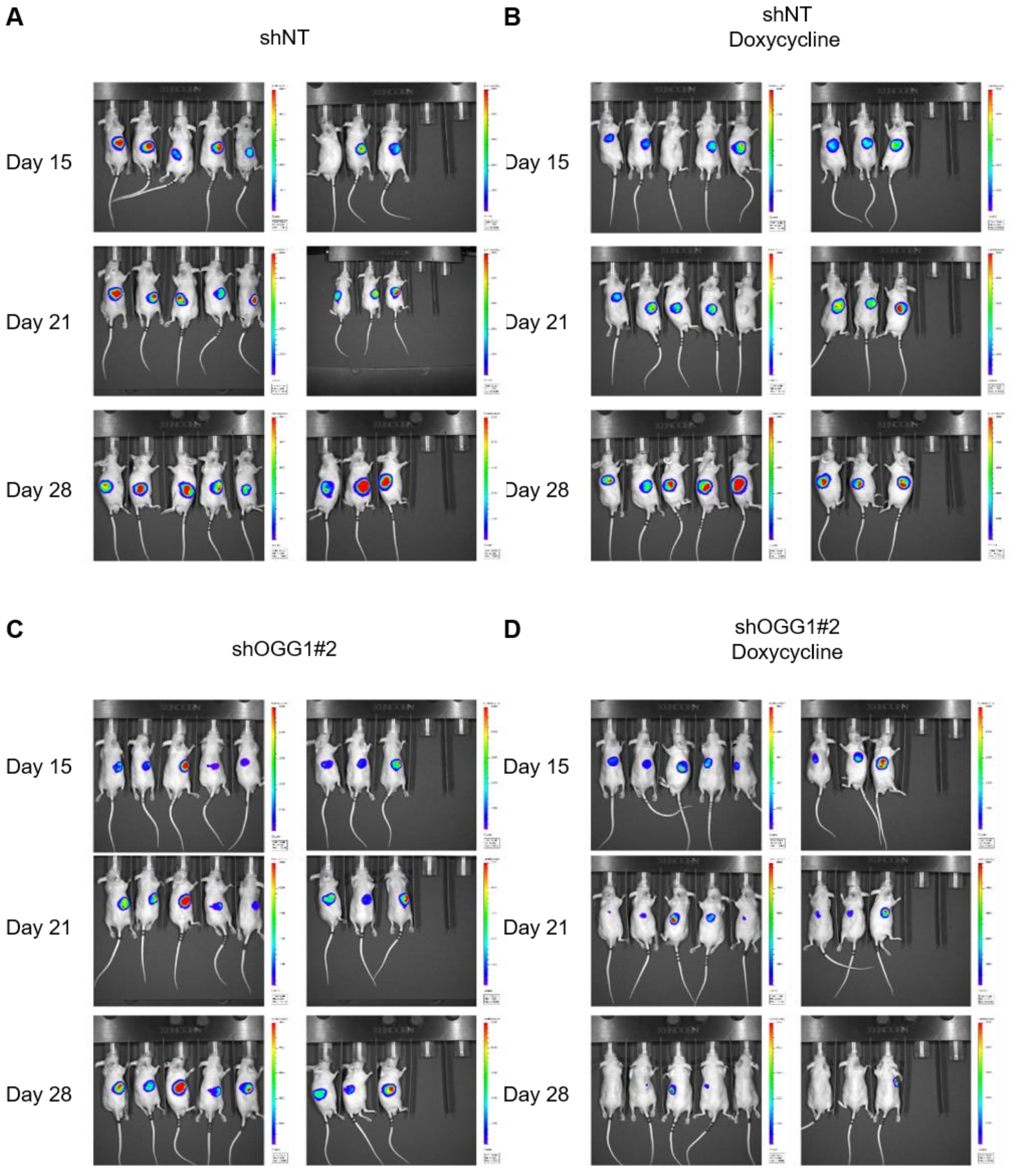

Supplementary Fig. S2: Bioluminescence imaging of A3 xenograft tumors. Ten million A3 cells stably expressing luciferase and the indicated doxycycline-inducible shRNA constructs were injected in mice and added doxycycline in the drinking water. Animals were imaged at the indicated times after doxycycline supplementation. **A.** Animals injected with a non-targeting shRNA construct. **B.** Animals injected with a non-targeting shRNA construct and supplemented with doxycycline in the drinking water. **C.** Animals injected with an *OGG1*-targeting shRNA construct. **D.** Animals injected with an *OGG1*-targeting shRNA constructs supplemented with doxycycline in the drinking water. The colours indicate intensity of bioluminescence, overlaid with a black and white photograph of the mice.

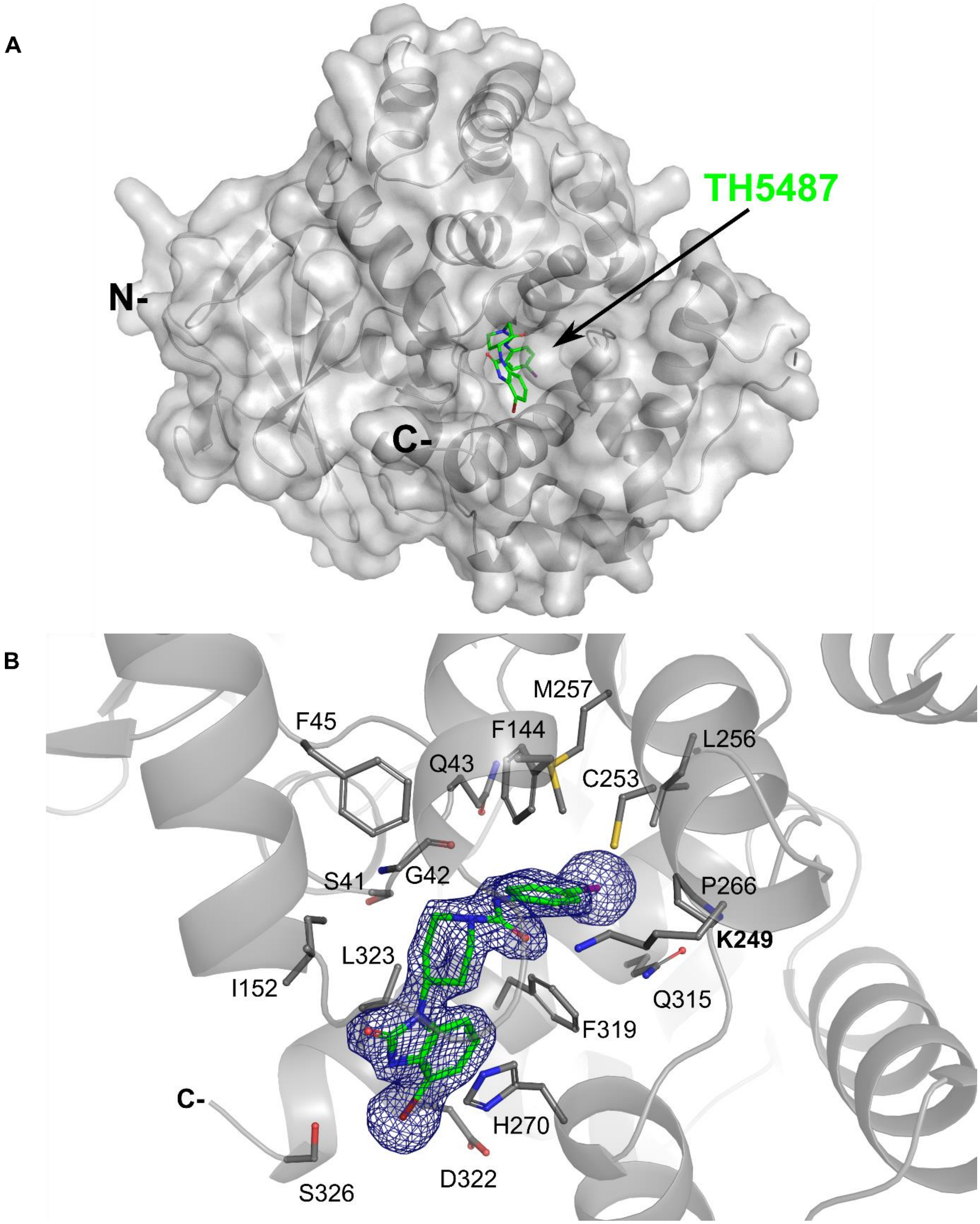

Supplementary Fig. S3: 'X-ray crystal structure of hOGG1 (grey) in complex with ligand TH5487 (green). **A.** Overall structure of human OGG1 in complex with TH5487. **B.** Close-up on the ligand-binding site. C-terminus, and important residues in the binding site are marked, electron density map around TH5487 shown as a blue mesh (2Fo-Fc map at 2σ level).

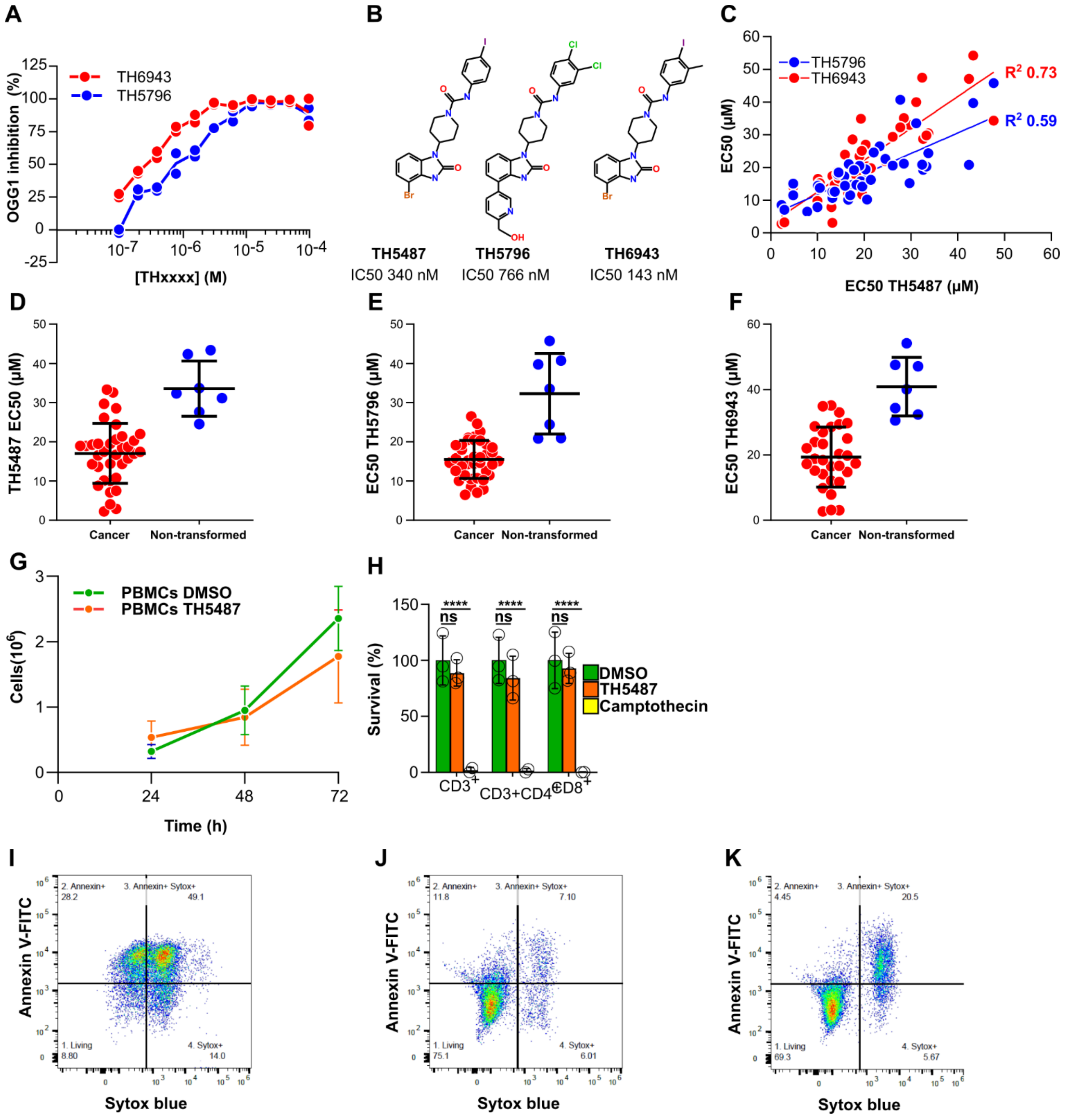

Supplementary Fig. S4: OGG1 inhibitors selectively inhibt cancer cell line proliferation A. TH5796 and TH6943 inhibit OGG1 activity *in vitro*. OGG1 enzyme was treated with the indicated concentration inhibitor in technical duplicates. The line connects the average values. TH5796 inhibits OGG1 with an IC50 of 766 nM, whereas the corresponding value for TH6943 is 170 nM.1 nM OGG1 was incubated with the indicated concentrations of TH5796 or TH6943 and 10 nM 8oxoA:C substrate in the presence of 2 nM APE1 as described. **B.** Chemical structure of TH5487 and the analogues described in this work. **C.** Correlation between TH5487 EC_{50} values and TH5796 or TH6943. Cell lines were treated for five days as in D, E, and F, and data are displayed as average EC50 values from 1–5 independent experiments. The lines indicate the best fit linear regression line, and R^2 the corresponding coefficient of determination. **D**. to **F.** Cancer cell selectivity for TH5487 (D), TH5796 (E) and TH6943 (F). Cell lines were treated as in Fig. 3E, and each point represents the EC50 value determined from 1–5 independent

experiments (Supplementary Table S1). **G.** TH5487 does not affect proliferation of activated PBMCs. PBMCs from two donors were activated using Dynabeads and exposed to 10 µM TH5487 during 72 h to follow cell numbers. **H.** TH5487 is well tolerated by T-cells. CD3+ T-cells were activated with Dynabeads and treated with DMSO or TH5487 for 6 days, or 25 µM camptothecin for 24 h as a technical control. Survival was assessed by flow cytometry. Data are three technical replicates from one donor, representative of four individuals. Paired, two-sided T-tests were used to assess significance (ns, non-significant, ****, $P<0.0001$). **I.** to **K.** Gating strategy for identification of dead cells. CD3+ cells were stained with Annexin V and Sytox Blue. Double-negative cells were designated as alive. The example plots show activated CD3+ cells treated with camptothecin (I), DMSO (J) and TH5487 (K).

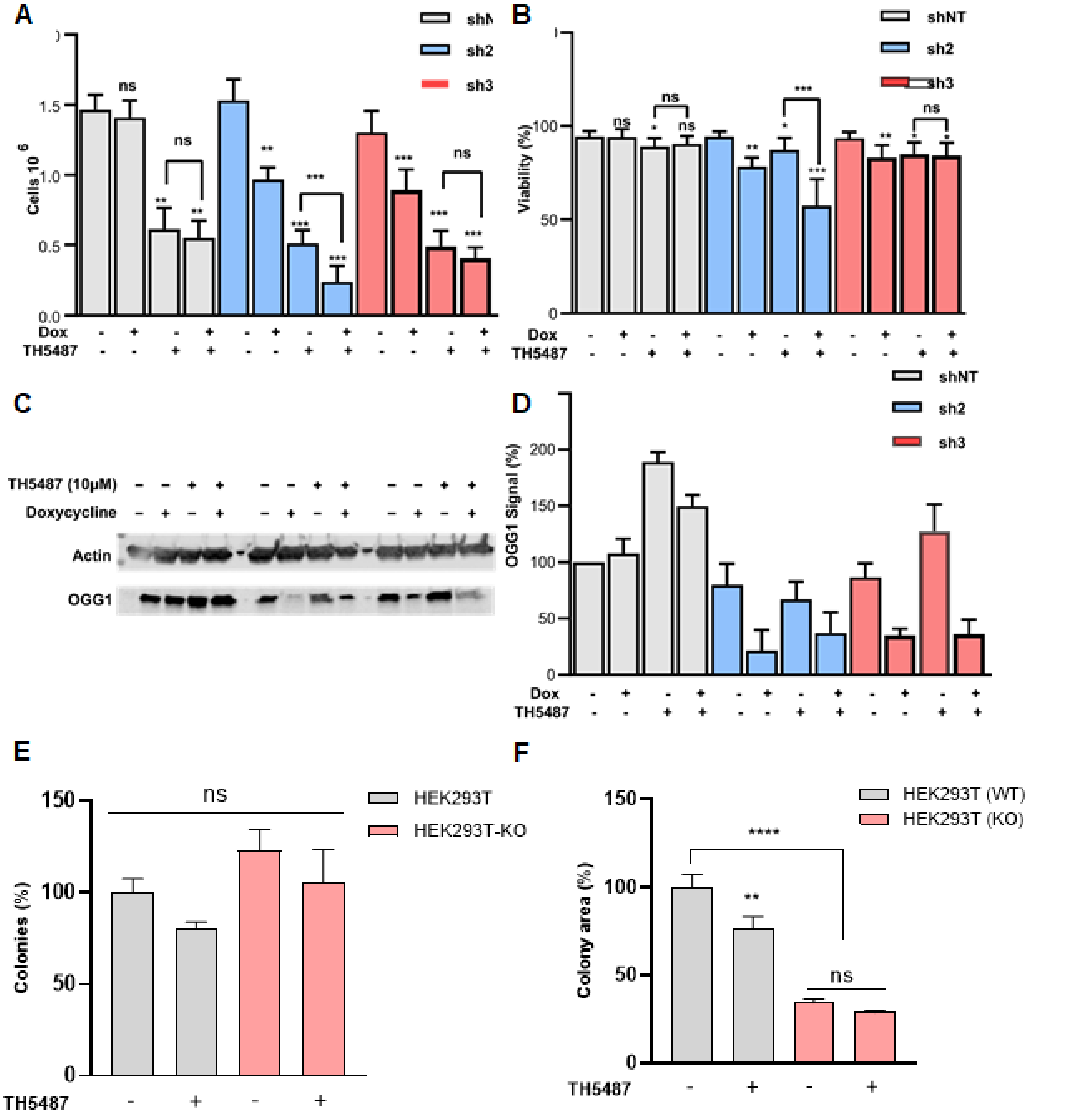

Supplementary Fig. S5: Off-target evaluation in A3 cells and HEK293T. A. Effects on proliferation of TH5487 in *OGG1* downregulated A3 cells. Cells were treated with doxycycline and/or 10 µM TH5487 for 96 h. Data are average ±SD of two technical replicates and are representative of three independent experiments. **B.** Effects on viability (living cells (%)) of TH5487 in *OGG1* downregulated cells. Data are average ±SD of two technical replicates and are representative of two independent experiments. Statistical significance was determined using unpaired, two-sided T-tests (ns, non-significant, *, P<0.05, **, P<0.01, ***, P<0.001 and ****, P<0.0001). **C.** Western blot showing OGG1 protein levels in A3 expressing shNT or sh2 and sh3 after doxycycline treatment, TH5487, or combination (doxycycline and TH5487). **D.** Comparative bar graph with quantification from two independent Western blots. **E.** Effect of TH5487 on colony formation in HEK293T cells. The number of colonies (%) is relative to that of

untreated parental HEK293T. Data are average ±SEM of 4 technical replicates from 2 independent experiments. **F.** Comparative analysis of the colony area (pixels) for HEK293T or HEK293T(KO) cells exposed for eight days to TH5487 or DMSO. Data are average ±SEM of 4 technical replicates from 2 independent experiments. Statistical significance was determined using Mann Witney test (**, P<0.01, ***, P<0.001 and ****, P<0.0001, ns, non-significant).

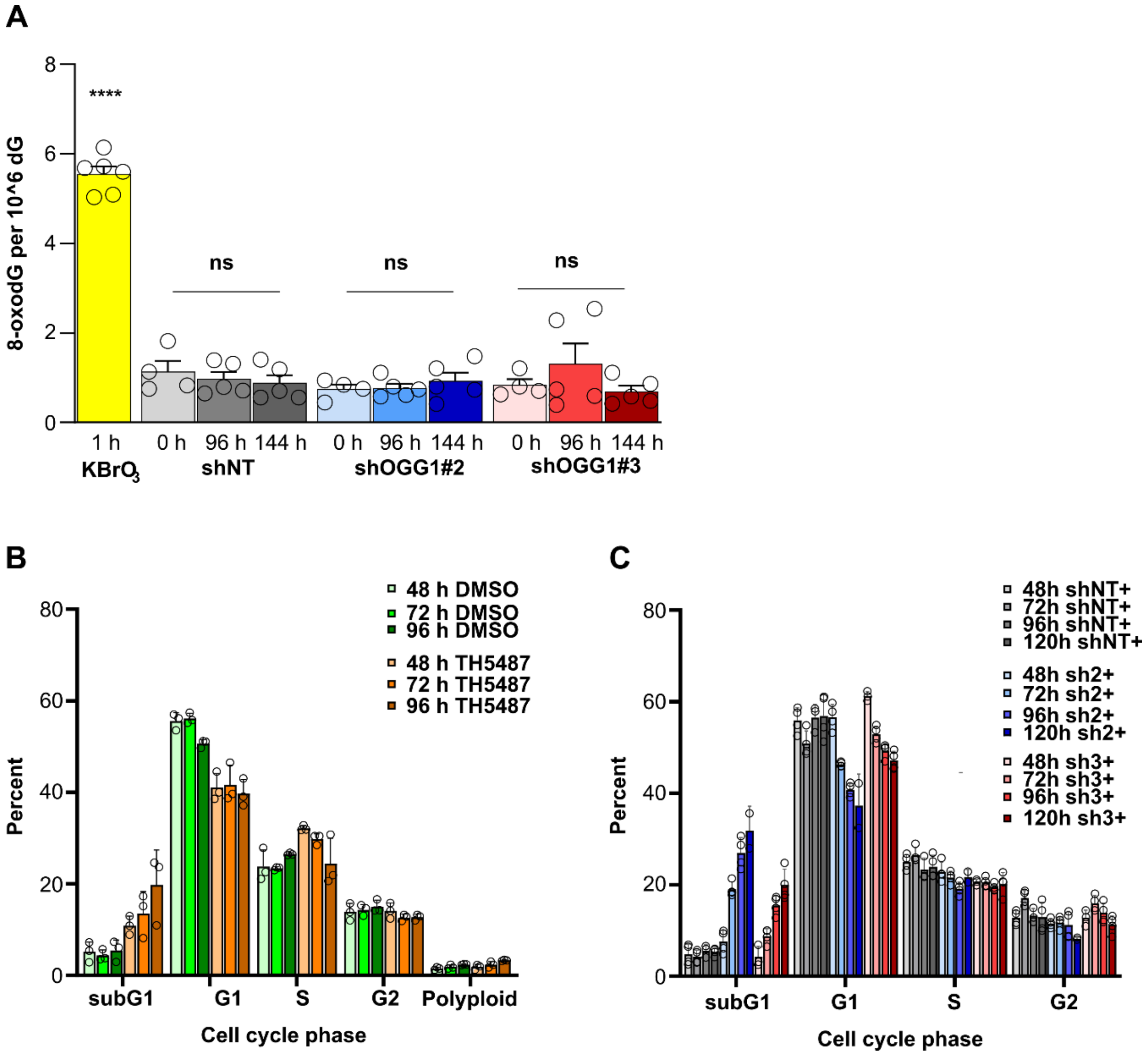

Supplementary Fig. S6: 8-oxodG levels and cell cycle distribution. A. 8-oxodG accumulation in A3 knockdown cells. The indicated shNT cells were treated with 250 ng/ml doxycycline for 0, 96, or 144 h, and the amount of genomic 8-oxodG was quantified with LC-MS/MS. As positive control, A3 shNT cells were treated with 20 mM $KBrO_3$ for 1 h (yellow bar). Data are average ±SD of five or six replicates from two independent experiments. **B.** Cell cycle distribution of OGG1 inhibited A3 cells. Cells were treated with 10 µM TH5487 or an equivalent volume DMSO for the indicated times, and the cell cycle distribution was determined by flow cytometry. Data points represent independent experiments (n=3). **C.** A3 shNT (grey), sh2 (blue), and sh3 (red), were treated with 250 ng/ml doxycycline for 48–120 h, and the cell cycle distribution was determined with flow cytometry. Data points represent technical replicates from two independent experiments. Statistical significance was determined using unpaired, two-sided T-tests (ns, non-signifant, ****, P<0.0001).

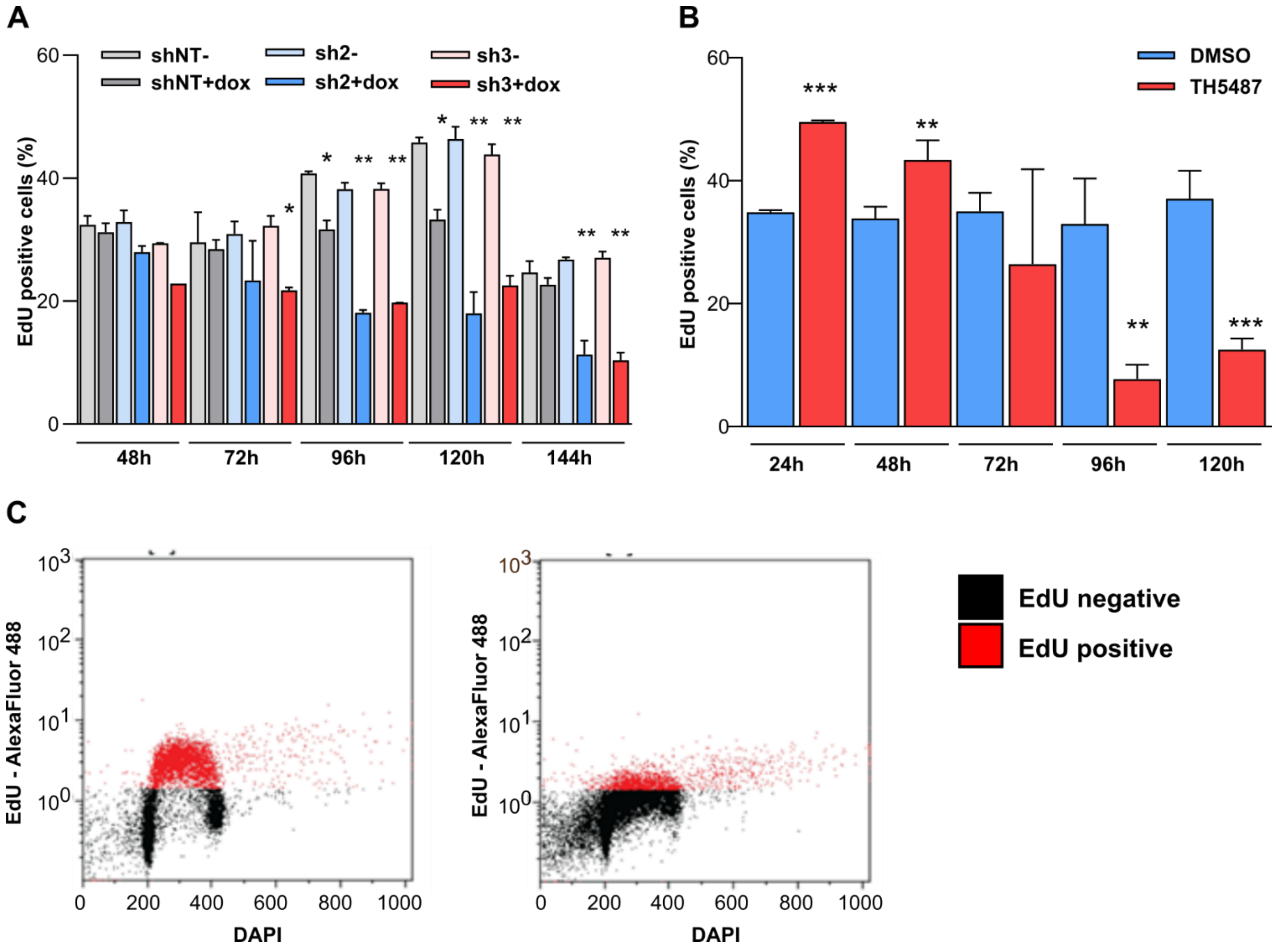

Supplementary Fig. S7: EdU incorporation in OGG1 perturbed A3 cells. A. Cells harboring the indicated shRNA constructs were treated with doxycycline for 48, 96 and 144 h and pulsed with EdU for 20 minutes before fixation, followed by Alexa-488 conjugation by click chemistry and quantitation by flow cytometry. OGG1 depletion caused a decrease in EdU positive cells 4 days after addition of doxycycline. Data are average ±SD of duplicate cultures, representative of two independent experiments. **B.** EdU incorporation in A3 cells treated with 10 µM TH5487. **C.** Gating strategy for identification of EdU positive cells. The dot plots indicate cells treated for 72 h with DMSO (left), and 10 µM TH5487 (right). In A and B, data are average ±SD of duplicate cultures representative of two independent experiments. Statistical significance was determined using unpaired, two-sided T-tests (*, P<0.05, **, P<0.01 and ***, P<0.001).

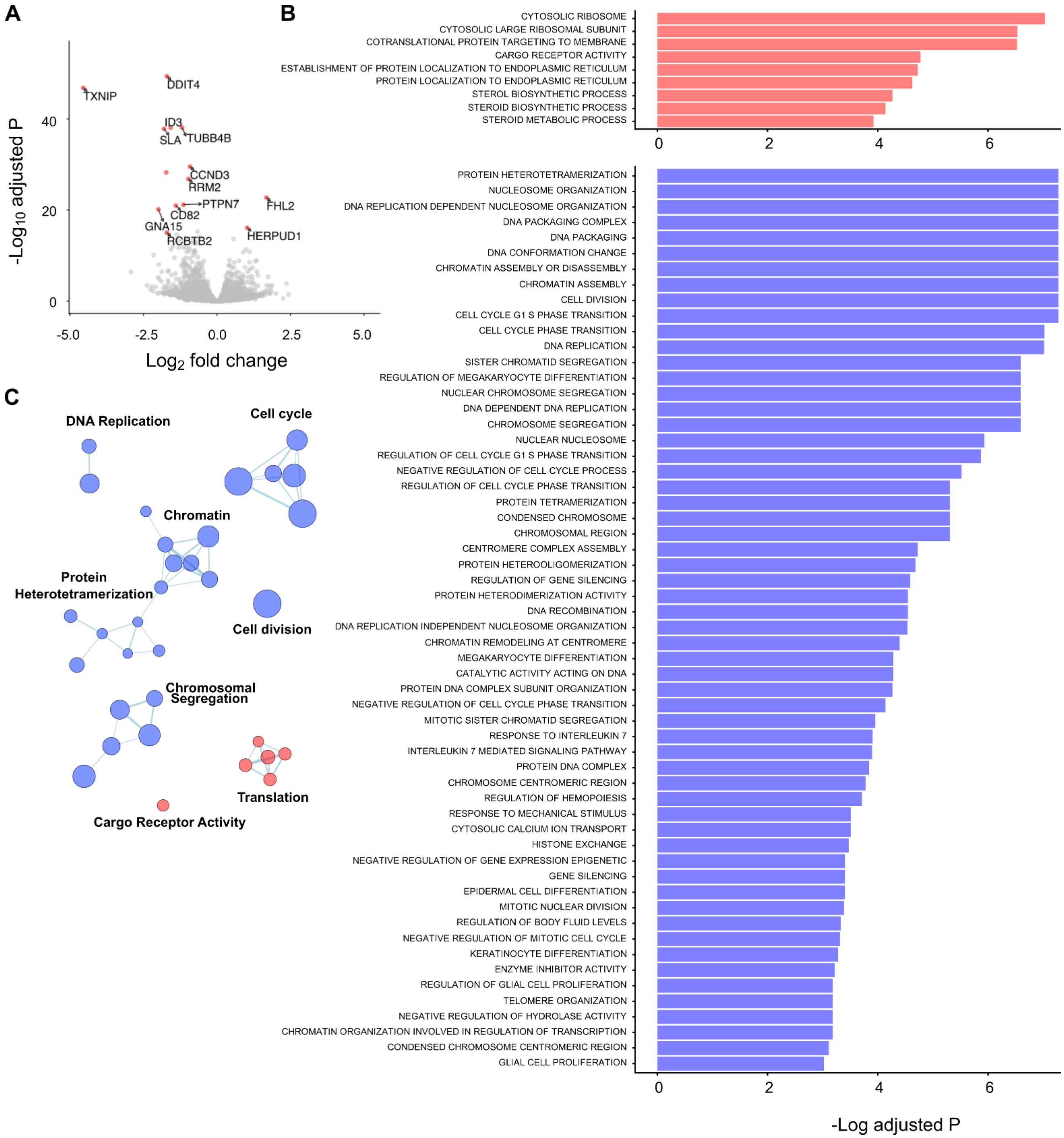

Supplemental Fig S8. Transcriptional changes induced by TH5487 treatment. A. Volcano plot displaying the transcriptional changes induced by 24 h of 10 µM TH5487. Transcripts with p<10e-15 and a log2 fold change of more than 0.75 are displayed in red. P values have been adjusted for multiple testing using the Benjamini-Hochberg method. **B**. Gene Set Enrichment Analysis (GSEA) results. Gene Ontology Gene Sets were taken from the MSigDB (C5: GO gene sets, v7.1). Upregulated gene sets are colored in red, downregulated ones in blue. Gene sets with a Benjamini-Hochberg adjusted P < 0.05 are displayed**. C.** Gene Ontology term enrichment map of the results of the GSEA. Nodes represent GO terms. Node size indicates the number of genes attributed to each GO term. Edges thickness indicates the amount of genes shared between two GO terms. Upregulated gene sets are colored in red, downregulated gene sets are colored in blue. Only GO terms with p<0.01 are shown. Clusters of common GO terms are named manually.

Supplementary tables

Supplementary Table S1: Effect of OGG1 inhibitors on cancer and non-transformed cell lines. Cell lines were incubated with a dilution series of the indicated compounds for 5 days, and viability assessed with resazurin. EC_{50}-values were calculated based on the resulting dose-response curves. The number of independent experiments is indicated for each value.

Cell line and Type C=cancer;N=non-transformed		Disease	TH5487		TH5796		TH6943	
			EC50 (µM)	n=	EC50 (µM)	n=	EC50 (µM)	n=
786-0	C	Kidney adenocarcinoma	**29.7**	2	**15.2**	2	**33.0**	2
A3	C	T-cell acute lymphoblastic leukemia	**10.0**	12	**7.9**	7	**9.8**	5
A498	C	Kidney carcinoma	**28.5**	2	**21.2**	2	**35.1**	2
ACHN	C	Kidney adenocarcinoma	**2.3**	3	**8.5**	1	**2.8**	3
BJ-Ras	C	RAS-transformed fibroblast	**4.8**	3	**15.1**	3	**14.1**	2
BJ-Tert	N	Tert-Immortalized fibroblast	**33.7**	3	**24.4**	3	**30.5**	2
CCD841	N	Non-transformed fibroblast	**43.3**	2	**39.7**	3	**54.2**	2
CCRF-CEM	C	T-cell acute lymphoblastic leukemia	**13.0**	5	**10.5**	2	**7.9**	2
Daudi	C	Burkitt's lymphoma	**16.6**	3	**10.1**	2	**12.1**	2
DU145	C	Prostate carcinoma	**13.2**	3	**10.7**	2	**3.1**	2
H460	C	Large cell lung carcinoma	**10.1**	3	**14.4**	3	**16.5**	3
HCT116	C	Colorectal carcinoma	**17.5**	3	**14.7**	3	**28.6**	2
HCT116+Chr3	C	Colorectal carcinoma with WT Chr3	**20.6**	2	**16.7**	2	**20.3**	2
Hec59	C	Endometrioid adenocarcinoma	**17.0**	2	**11.5**	2		
Hec59+Chr2	C	Endometrioid adenocarcinoma with WT Chr2	**15.7**	2	**17.3**	2		
HeLa	C	Cervix adenomcarcinoma	**19.3**	3	**15.8**	2	**34.9**	2
HL-60	C	Acute promyelocytic leukemia	**15.9**	4	**14.4**	1	**23.9**	1
HT-29	C	Colorectal adenocarcinoma	**24.5**	3	**22.6**	3	**22.0**	1
Jurkat	C	T-cell acute lymphoblastic leukemia	**10.5**	5	**13.8**	2	**15.2**	2
K562	C	Chronic myelogenous leukemia	**16.7**	5	**21.0**	4		
KG1a	C	Acute myelogenous leukemia	**14.3**	2	**18.0**	1	**18.8**	1
LCL#1	N	Non-transformed lymphoblastoid cell line	**31.1**	5	**33.5**	4	**40.0**	2
LCL#2	N	Non-transformed lymphoblastoid cell line	**27.7**	5	**40.7**	4	**32.3**	3
M059J	C	Glioblastoma	**26.1**	3	**20.6**	3	**29.4**	3
M059K	C	Glioblastoma	**20.3**	4	**19.4**	4	**27.0**	4
MCF7	C	Breast adenocarcinoma	**23.3**	2	**26.5**	3		
MEF *Ogg1*(-/-)	N	Non-transformed mouse embryonic fibroblast	**47.7**	4	**45.8**	3	**34.3**	3
MOLT4	C	T-cell acute lymphoblastic leukemia	**13.2**	6	**12.6**	2	**17.4**	3
MRC5	N	Non-transformed lung fibroblast	**42.4**	2	**20.8**	2	**47.1**	2
MV4-11	C	Acute monocytic leukemia	**7.8**	5	**6.5**	2	**9.6**	2
NB-4	C	Acute promyelocytic leukemia	**4.8**	5	**11.5**	3		
PC3	C	Prostate adenocarcinoma	**32.6**	4	**19.3**	2	**28.7**	3
PL-21	C	Acute myeloid leukemia	**20.6**	6	**10.2**	3	**18.4**	3
Raji	C	Burkitt's lymphoma	**19.0**	4	**20.5**	2	**23.4**	2
Rec1	C	B cell non-Hodgkin's lymphoma	**13.7**	4	**12.6**	2	**14.8**	3
Reh	C	Acute lymphocytic leukemia (non-T, non-B)	**18.3**	3	**14.7**	3	**17.3**	2
RFX393	C	Kidney carcinoma	**19.6**	2	**14.2**	2	**11.7**	2
SaOS2	C	Osteosarcoma	**21.4**	3	**16.2**	3	**19.8**	3
SW1271	C	Small cell lung carcinoma	**22.0**	2	**24.6**	2		
SW480	C	Colorectal adenocarcinoma	**33.3**	3	**20.3**	4	**29.8**	2
T98G	C	Glioblastoma	**14.5**	2	**18.5**	2	**16.7**	2
THP1	C	Acute monocytic leukemia	**17.8**	4	**19.2**	2		
U2OS	C	Osteosarcoma	**19.5**	5	**16.1**	4	**25.1**	2
UO31	C	Kidney carcinoma	**2.9**	3	**7.1**	3	**3.2**	3
VH10	N	Non-transformed fibroblast	**32.4**	2	**20.9**	2	**47.5**	1

Supplementary Table S2: List of primers for used in this study (Figure 6F)

Gene	Sequence
MCM7-F	ACCGAGACAATGACCTAC
MCM7-R	GCTATGTAACGCCTCATG
MCM6-F	ACTGTTCCTGGACTTCTTGG
MCM6-R	ACGAATCAGTTCCTCTGCTAAT
MCM5-F	GTTTGACAAGATGCGAGAA
MCM5-R	CCTTGGCGATAGAGATGG
MCM2-F	AGGCAGCATCCCCATTAC
MCM2-R	TCACATAGTCCCGCAGAT
FEN1-F	GCCAATCCAGGAATTCCACC
FEN1-R	GATTCGCTCCTCAGAGAACTGCTT
MCM3-F	GAGTGAATCCAGGTTGAAGG
MCM3-R	GATTCTGTGAGGCGATTCAT
PCNA-F	CCATCCTCAAGAAGGTGTTGG
PCNA-R	ATAAAATTGCGGATATGGGACAC
MCM4_F	TCACCACTGACATACGGCAC
MCM4_R	GTGTGCCCCTAACACCACTT

Supplementary Table S3: X-ray crystallography – data collection and refinement statistics. [a] Values in parentheses are for highest-resolution shell. [b] Values for each molecule of the asymmetric unit.

	**hOGG1:TH5487**
**Data collection**	
PDB code	6RLW
Station	DLS-I04
Space group	$P4_12_12$
Cell dimensions	
a, *b*, *c* (Å)	86.4, 86.4, 432.4
α, β, γ (°)	90.0, 90.0, 90.0
Resolution (Å)	2.00-86.4 (2.00-2.03)[a]
No. total / unique reflections	2,446.343 / 112,319
R_{merge}	0.096 (1.22)[a]
R_{pim}	0.029 (0.39)[a]
$CC_{1/2}$	1.00 (0.85)[a]
$I / \sigma I$	16.5 (2.7)[a]
Completeness (%)	100.0 (99.9)[a]
Redundancy	21.8 (20.6)[a]
Wilson B factor	32.6
**Refinement**	
No. reflections used	106,369
R_{work} / R_{free} (%)	23.0 / 27.6
B-factors	
Protein (all atoms)[b]	38.1 / 40.1 / 47.3 / 53.4 / 45.4
Ligand[b]	32.1 / 51.4 / 68.0 / 67.7 / 45.3
Water	45.2
R.m.s. deviations	
Bond lengths (Å)	0.005
Bond angles (°)	1.29
Ramachandran statistics	
Favored (%)	95.8
Outliers (%)	0.00
